# Distinct repair processes produce APOBEC-induced deletions, tandem substitutions, and complex mutations in yeast and human cells

**DOI:** 10.1073/pnas.2609794123

**Published:** 2026-07-28

**Authors:** Alexandra Puzon, Atri Raval, Cameron Cordero, Daniel Schiefen, Piotr A. Mieczkowski, Steven A. Roberts, Tony M. Mertz

**Affiliations:** ^a^ School of Molecular Biosciences and Center for Reproductive Biology, Washington State University, Pullman, WA 99164; ^b^ Department of Microbiology and Molecular Genetics, University of Vermont, Burlington, VT 05405; ^c^ Department of Genetics, Lineberger Comprehensive Cancer Center, University of North Carolina, Chapel Hill, NC 27599

**Keywords:** APOBECs, DNA repair, cancer, mutagenesis

## Abstract

APOBEC3A (A3A) and APOBEC3B (A3B) induce single base substitution signatures SBS2 and SBS13, which are composed of C>T and C>G mutations, respectively, at TCW sequences in >50% of sequenced tumors. However, few driver mutations have been attributed to APOBEC-induced SBS mutations. Although SBS2 and SBS13 have been associated with additional mutation types, the contribution of APOBECs to non-SBS mutations and the mechanisms that generate noncanonical APOBEC-induced mutations are uncharacterized. Here, we show that A3A expression generates non-SBS mutations including C deletions within TTC motifs, CC>TT and TC>AT/CT/GT dinucleotide variants (DNVs), distinct classes of paired SNV-SNV events, and complex insertion-SBS mutations in a yeast model system. Furthermore, we found that endogenous APOBEC expression in breast cancer cell lines is a significant driver of all these mutation types. In addition, we show that C deletions and complex insertion-SBS mutations within TTC motifs occur primarily by strand slippage during translesion synthesis (TLS) bypass of APOBEC-induced, UNG-dependent, abasic sites. Additionally, we found that CC>TT and TC>AT/CT/GT DNVs, which comprise the APOBEC-associated DBS11 signature, are generated by dU templating and TLS bypass of APOBEC-induced abasic sites, respectively. We also present data indicating that phased APOBEC-induced SNV-SNV mutations are produced during abasic site bypass by TLS. Analysis of WGS data from cultured human cells and tumors indicates that similar mechanisms generate these noncanonical APOBEC-induced mutations in human cancers, providing additional means by which APOBECs could contribute to carcinogenesis and therapeutic resistance.

## Significance

Dysregulated APOBEC activity is one of the most prominent sources of base substitution mutations in human cancer. Here, we present evidence supporting seven mechanisms by which APOBECs induce non–single base substitution mutations, including deletions, doublet substitutions, and additional complex events. These findings expand the repertoire of mutations caused by APOBECs. Identified mechanisms involve differential processing of APOBEC-induced damage, providing insight into mechanisms of lesion bypass. These additional APOBEC-induced mutation types expand the potential paths by which APOBECs drive tumorigenesis and therapeutic resistance.

Activation Induced Cytidine Deaminase (AID)/Apolipoprotein B mRNA editing enzyme, catalytic polypeptide-like (APOBEC) proteins are a family of cytidine deaminases that promote immunoglobulin diversification (1) and restrict virus and retrotransposon replication (2). The APOBEC3 subfamily employs multiple mechanisms to restrict intracellular foreign nucleic acids including deamination of deoxycytidines (dC) to deoxyuridines (dU) in single-stranded (ss) DNA (3, 4). APOBEC3A (A3A), APOBEC3B (A3B), APOBEC3C, and APOBEC3H can enter the nucleus (5–7), enabling them to deaminate genomic DNA (7–15) in addition to foreign DNA substrates. Enzymatic activity (16, 17), mesoscale features (18), YTCA or RTCA preference (19), and CRISPR/Cas9 deletion analyses (20) indicate that A3A and A3B cause the majority of COSMIC single base substitution (SBS) signatures 2 and 13 (i.e., C>T or C>G mutations in TCW motifs, respectively, where W is an A or T), which are observed in over 50% of whole genome sequenced cancer genomes (21, 22). These APOBEC-induced substitutions are responsible for a small subset of cancer-causing mutations in human tumors (23–25). However, humanized mouse models indicate that A3A and A3B can promote cancer initiation (26, 27) and therapeutic resistance (28–30) through SBS-independent mechanisms. Several types of non-SBS APOBEC mutagenesis have been reported that could contribute to these cancer phenotypes.

We found significant numbers of C deletions and additional complex insertion-single base substitution mutations in TC sequence contexts in yeast strains expressing A3B and A3A (31). Another study reported that A3A overexpression in avian DT40 cells generates C deletions in TC contexts and that similar deletions correlate with SBS2/13 mutations in human tumors (32). In addition to indels, COSMIC doublet base substitution (DBS) signature DBS11, which is primarily composed of CC>TT mutations, has been associated with APOBEC signatures SBS2 and SBS13 (21). APOBECs also generate closely spaced SBS mutations termed *didyma* (Greek for twins), which may encompass several types of events with different underlying molecular mechanisms (25, 33). However, beyond these correlative associations little is known about the propensity of APOBECs to induce indels, DBS signature mutations, and other complex mutation types and the molecular mechanisms responsible for these events are unknown.

DNA repair pathways play significant roles in preventing and diversifying the spectrum of APOBEC-induced mutations. SBS2 can result from either templated bypass of APOBEC-induced dU or A-rule insertion and Polζ-mediated extension past abasic sites generated by dU removal by an uracil-DNA glycosylase, yeast Ung1 or human UNG2. In addition, SBS13, requires Ung1/UNG2 to convert dU to an abasic site and C-rule insertion by translesion synthesis (TLS) enzyme Rev1 and extension by Polζ (20, 34, 35). Conversely, data from yeast (31, 34, 36) and DT40 (37–39) models and human tumors (31) indicate homologous recombination (HR)-dependent lesion bypass reduces mutations derived from APOBEC-dependent abasic sites. Thus, the production of non-SBS APOBEC-induced mutations is also likely modulated by dU removal and multiple DNA repair pathways.

Here, we utilized novel mutation reporters, whole genome sequencing (WGS), and bioinformatics analysis of sequenced tumor data to define mechanisms that produce APOBEC-induced indels, DBS signatures, and complex mutations. We found that A3A and A3B (A3A/B) expression generates C deletions and T insertions 5′ of APOBEC signature C>T and C>G SBSs in TTC motifs and that these events are generated by stand slippage during TLS bypass of UNG-dependent abasic sites in both yeast and cultured human cells. We demonstrate that the primary components of DBS11, CC>TT mutations and TC>AT/CT/GT mutations, occur by sequential dU templated synthesis and TLS-dependent bypass of APOBEC/UNG-dependent abasic sites, respectively. In addition, we demonstrate that *didyma*-like, A3A/B-induced paired SNVs can be generated by Ung1/UNG2-dependent TLS bypass of abasic sites. We also show that these mutation types correlate with SBS2/13 abundance in human tumors. As noncanonical APOBEC-induced mutations are likely to produce more severe changes, they could play outsized roles in the inactivation of tumor suppressor genes, restoration of BRCA1 and BRCA2 function, which provides resistance to PARP inhibitors (40, 41), and generation of strong neoantigens to be exploited clinically (42, 43).

## Results

### APOBEC Cytidine Deaminases Increase the Rate of Indel Mutations.

**Fig. 1.**
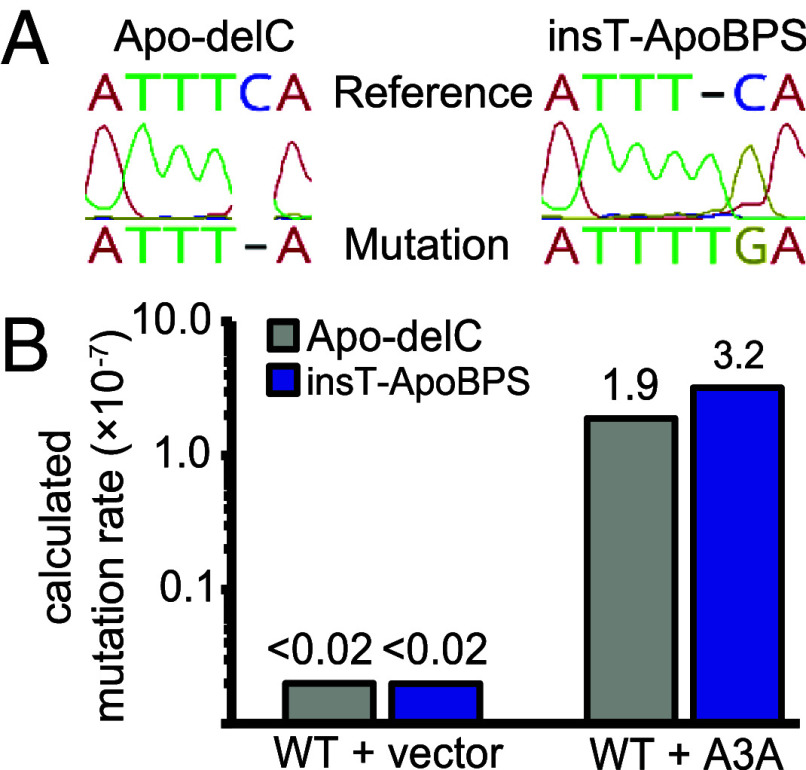
APOBEC expression generates specific indel mutations in TC contexts in yeast. (*A*) *CAN1* mutations previously overserved in BY4741 *mph1*Δ yeast expressing A3B (31). The *CAN1* reference sequence is on *Top* and mutant sequences are on the *Bottom*. (*B*) Apo-delC (gray) and insT-ApoBPS (blue) mutation rates in WT BY4741 yeast with either a control vector or plasmid expressing A3A were calculated by multiplying the observed mutation rate times the fraction of mutations for each mutation type. Data from Supplemental Data 1. Data labels display mutation rate values. Note: For WT + vector, these mutation types were not observed in 63 sequenced *CAN1* mutants and the rate was estimated from a fraction of 1/63.

We previously identified 2938 independent *CAN1* mutations in yeast strains containing genetic defects and expressing A3B (31) and found that approximately 6% of all the mutations were deletions of C in TC motifs (Apo-delC mutations) and T insertions 5′ of APOBEC signature C>T and C>G SBSs (insT-ApoBPS mutations) (Fig. 1*A*). To quantify APOBEC-induced indels in a wild-type (WT) yeast strain, we sequenced *CAN1* mutations in WT BY4741 yeast with an A3A expression vector or empty vector control. A3A expression increased the mutation rate 141-fold and ~99% of all mutations occurred in TC sequence context (Dataset S1). 89% of indels in A3A expressing yeast occurred in APOBEC-target TC sequences. Although Apo-delC and insT-ApoBPS only comprised 0.9% and 1.4% of the APOBEC induced mutations, these mutation types were not observed in yeast without A3A expression and are elevated >91-fold and >152-fold, respectively, in A3A expressing yeast (Fig. 1*B* and Datasets S1 and S2).

**Fig. 2.**
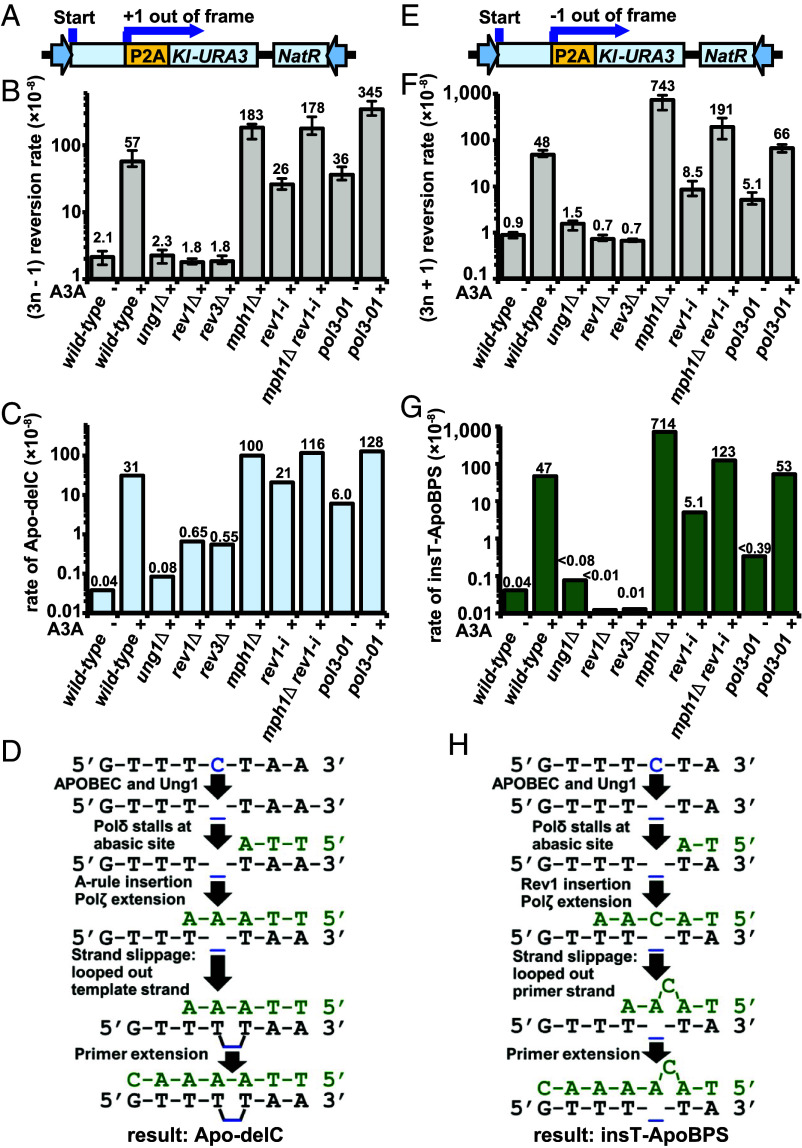
APOBEC-induced indels occur due to slippage events during TLS past *UNG1*-dependent abasic sites. (*A*) A reversion reporter composed of the *PDA1* promoter, start codon, a reversion track derived from *CAN1* that contains five hotspots for APOBEC-induced indels observed from (31), which places the downstream CDS+1 out of frame, followed by a P2A ribosome skip site encoding sequence, and the *URA3* gene from *Kluyveromyces lactis*. Adjacent to the reversion reporter sequence is the nourseothricin resistance gene (NatR), which allows for selection of yeast with the integrated reporter. The reporter is integrated 522 bp upstream of *CAN1* in the 5′ to 3′ orientation. (*B*) The observed *URA3* reversion rate for (3z−1) base pair indels, where z is any integer. Error bars represent 95% CI and data labels display the mutation rate (×10^−8^). (*C*) Rates were calculated by multiplying the fraction of mutation spectra composed of Apo-delC mutations by the reversion rate for each genotype. (*D*) A model for Apo-delC mutations in TTC context. Black font represents the lagging strand template during DNA replication. Green font represents newly synthesized DNA. The position of the cytidine deaminated by A3BA/A3B is in blue font. (*E*) Same as Fig. 2*A*, but the length of the reversion track places the P2A element and *URA3* minus 1 bp out of frame. (*F*) As in Fig. 2*B*, but for the observed *URA3* reversion rate for (3z+1) base pair indels, where z is any integer. (*G*) Rates were calculated by multiplying the fraction of mutation spectra composed of insT-ApoBPS mutations by the reversion rate for each genotype. (*H*) A model for insT-ApoBPS in TTC context. Denotation of strands as in panel *D*. In panels *B*, *C*, and *F*, and *G* *rev1*-*D467A, E468A* is denoted as *rev1-i*, with “i” indicating an inactive dC transferase.

To characterize the mechanisms responsible for Apo-delC and insT-ApoBPS mutations, which comprise a small fraction of total mutations, we generated reversion mutation reporters capable of observing 3z+1 and 3z−1 frameshifts (where z = integers) within a 933 bp reversion track that contains five hotspot sequences for APOBEC-induced indels from our previous *CAN1* mutation spectra. Utilizing these reversion reporters (Fig. 2 *A* and *E*), we found that A3A expression significantly increased the observed rate of both 3z+1 and 3z−1 frameshifts (Fig. 2 *B* and *F* and Dataset S2). By multiplying observed reversion rate by the fraction of the mutation spectra composed of specific events (Dataset S2), we estimated the mutation rate for specific mutation types. We found that A3A expression increases the rate of Apo-delC 808-fold and complex insT-ApoBPS 1122-fold (Fig. 2 *C* and *G* and Datasets S1 and S2). Apo-delC and insT-ApoBPS mutations were also increased by A3B expression in these reporter strains (*SI Appendix*, Fig. S1 and Datasets S1 and S2). In addition to Apo-delC mutations, we also observed insertions of two nucleotides, either TT or CT, immediately 5′ of C>D (D = A, T, and G) substitutions in TC motifs, which comprised 21% of mutations observed with the 3z −1 reporter in WT yeast expressing A3A. Most of these events occurred in a CTCTCTCA sequence context. Moreover, nearly all Apo-delC and insT-ApoBPS mutations occurred at sequences with ≥3 T nucleotides 5′ the APOBEC target cytidine (Dataset S1 and *SI Appendix*, Figs. S2 and S3), suggesting these types of APOBEC-induced mutations are facilitated by flanking dinucleotide and mononucleotide repeats and therefore likely occur via stand slippage mechanisms.

### Apo-delC and insT-ApoBPS Occur by Strand Slippage Following TLS Bypass of Abasic Sites Generated by Ung1.

To determine the contribution of DNA repair pathways to Apo-delC and insT-ApoBPS mutations, we measured the rate of A3A-induced 3z−1 and 3z+1 frameshifts in reporter strains with deletions of *UNG1*, *REV1*, *REV3* (the catalytic subunit of Polζ). We found that the reversion rates for *ung1Δ*, *rev1Δ*, and *rev3Δ* strains expressing A3A were similar to the WT yeast without A3A expression (Fig. 2 *B* and *F*). Additionally, Apo-delC and insT-ApoBPS mutations were decreased greater than 47-fold and 587-fold, respectively, in *ung1Δ*, *rev1Δ*, and *rev3Δ* yeast, compared to respective WT strains expressing A3A, (Fig. 2 *C* and *G*) and thus both types of mutations occur primarily during TLS bypass of abasic sites. We also found that strains lacking *MPH1*, a DNA helicase required for the bypass of lesions by template switching, which suppresses TLS mutagenesis (44), displayed elevated rates Apo-delC and insT-ApoBPS events (Fig. 2 *C* and *G*), further implicating TLS in the formation of these mutation types.

To further parse the mechanism(s) responsible for APOBEC-induced indels, we utilized a hypomorphic *REV1* mutation, *rev1-D467A, E468A* that eliminates the deoxycytidyl transferase activity of Rev1, while maintaining the ability of Rev1 to scaffold for other polymerases, which is required for TLS (45). The *rev1-D467A, E468A* mutation did not decrease the rate of Apo-delC mutations, which indicates that A-rule incorporation is responsible for Apo-delC mutations. Hence, we propose that the primary mechanism driving Apo-delC mutations is Polζ extension from a dA inserted opposite the abasic site into the 5′ T homopolymer prior to template strand slippage, which would generate a primer terminus stabilized by ≥2 A-T base pairs post–strand slippage (Fig. 2*D*).

Because most APOBEC-induced mutations occur due to deamination of the lagging strand template (15, 46), Polδ may be responsible for dA insertion during abasic site bypass. Polδ can insert an A opposite an abasic site but is inefficient at extending the mismatched primer terminus and thus requires Polζ for extension (47). Pol3, the catalytic subunit of yeast Polδ, also possesses a 3′ exonuclease activity (48), which can remove the unpaired A at the primer terminus, potentially limiting A-rule bypass. We measured the rate of Apo-delC mutations in *pol3-01* yeast, which lack Polδ exonuclease activity (49) and found that loss of exonuclease activity synergistically increases Apo-delC mutations when combined with A3A expression (Fig. 2*C*). Additionally, Apo-delC mutations in *pol3-01* yeast occurred in both typical hotpots (i.e., TTC sequence motifs) and additionally within DTC motifs (*SI Appendix*, Fig. S2), which indicates that another mechanism not requiring the 5′ T homopolymer also functions when the inserted A opposite the abasic site is stabilized. In addition, the rates of Apo-delC mutations in *rev1Δ* and *rev3Δ* strains expressing A3A were significantly higher than that of WT yeast without A3A and *ung1Δ* yeast with A3A (Fig. 2*C*). The A3A-induced Apo-delC mutations in *rev1Δ* and *rev3Δ* yeast also occurred at both TTC and DTC sequence contexts (*SI Appendix*, Fig. S2). The prevalence of C deletions in DTC contexts in *pol3-01* and TLS deficient yeast indicate that an A-rule insertion, slippage, and extension mechanism (*SI Appendix*, Fig. S4*B*) can also generate Apo-delC mutations.

Approximately 89% of the BPS within insT-ApoBPS events in WT A3A-expressing yeast were C>G (Dataset S1), which suggests Rev1 catalytic activity plays a key role in their formation. Also, the rate of insT-ApoBPS mutations was reduced ninefold (Fig. 2*G*) in *rev1-D467A, E468A* yeast, indicating a strong dependence on C insertion by Rev1. Additionally, the rate insT-ApoBPS with a C>T (or A) BPS (i.e., excluding events with C>G BPS) was 4.7 × 10^−8^ and 5.1 × 10^−8^ for *rev1-D467A, E468A* and WT A3A expressing yeast, respectively. Also, the rate of insT-ApoBPS mutations in *pol3-01* yeast expressing A3A was not increased compared to WT with A3A (Fig. 2*G*). The failure of *rev1-D467A, E468A* and *pol3-01* alleles to elevate insT-ApoBPS mutations containing C>T/A substitutions indicates that A-rule bypass inefficiently generates insT-ApoBPS mutations, or that Apo-delC mutations are heavily favored over insT-ApoBPS during A-rule bypass. Together these results indicate that insT-ApoBPS mutations occur primarily due to Rev1 and Polζ bypass of APOBEC/UNG-generated abasic sites and subsequent slippage of the primer strand (Fig. 2*H*). We also measured insT-ApoBPS mutations at a naturally occurring +1 reporter, *his7-2*, in a different yeast genetic background for WT, *Δung1*, *Δrev3*, *Δmph1, and rev1-D467A, E468A* yeast strains expressing A3B. These results using the *his7-2* reversion reporter (*SI Appendix*, Fig. S5 and Datasets S1 and S2) mirrored those generated with the *URA3* reversion reporter (Fig. 2), which indicates our findings are not an allele or strain specific phenomenon.

### Nonbiased APOBEC-Induced Mutation Spectra from WGS.

**Fig. 3.**
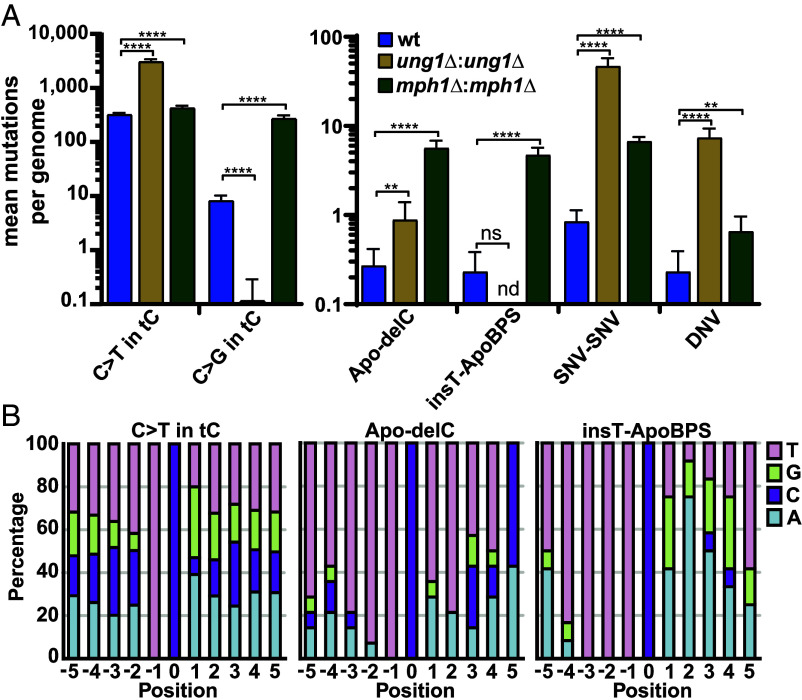
Genome wide APOBEC-induced indels and complex events are modulated by Ung1 and bypass pathways. (*A*) The mean frequency of APOBEC-induced mutations per genome from WGS of serially passaged yeast strains (error bars denote 95% CI). 52, 17, and 29 genomes were sequenced for WT, *ung1*Δ/*ung1*Δ, and *mph1*Δ/*mph1*Δ yeast. For complex mutation types, insT-ApoBPSs, SNV-SNV events, and DNVs, only phased mutations were included in the analysis. ***P < 0.001 and ****P < 0.0001 based on pairwise Mann–Whitney *U* tests. Not Significant (ns). Not Detected (nd). (*B*) Sequence context of given mutation types.

To determine the frequency of APOBEC-induced indels unbiased by limited sequence contexts and selection, we performed WGS on diploid yeast strains expressing A3A that were each propagated for approximately 625 population doublings to accumulate mutations. We observed 18486 total SNVs, of which only 186 occurred at an A or T reference bases compared to 18363 SNVs at C or G reference bases, ~94% of which were C>T or C>G in TC sequence contexts, indicating that nearly all the observed mutations were A3A-induced. Consistent with previous studies (31, 34), C>T SBS in TC motifs were increased by strains lacking Ung1 while C>G SBS in TC motifs were decreased 71-fold and increased 33-fold in *ung1Δ*/*ung1Δ* and *mph1Δ*/*mph1Δ* yeast, respectively (Fig. 3).

Apo-delC mutations comprised 67%, 94%, and 91% of all deletions at C and G reference bases in WT, *ung1Δ*/*ung1Δ,* and *mph1Δ*/*mph1Δ* yeast expressing A3A, respectively (Dataset S3). As observed with our reversion reporter and consistent with our model (Fig. 2*D*), the frequency of Apo-delC mutations per genome was increased in *mph1Δ*/*mph1Δ* yeast. Contrary to results observed with our reversion reporter (Fig. 2*C*), the number of Apo-delC mutations per genome was ~3.3-fold higher for *ung1Δ*/*ung1Δ* compared to WT yeast (Fig. 3 and Datasets S3 and S4). To gain insight into why Apo-delC mutations are increased genome wide in *ung1Δ*/*ung1Δ* yeast, we examined the mean T homopolymer length interrupted by the deleted C nucleotide. We found that interrupted T homopolymer lengths appeared to have a bimodal distribution for WT (mean length 7.14 bp) and *mph1Δ*/*mph1Δ* yeast (mean length 5.54 bp) with peaks at 3 to 4 repeats and 9 to 12 repeats (*SI Appendix*, Fig. S6). In contrast, Apo-delC events in *ung1Δ*/*ung1Δ* yeast occurred primarily in long interrupted T homopolymers (mean length = 10 repeats). We hypothesize that the increase in Apo-delC mutations in *ung1Δ*/*ung1Δ* relative to WT is due to conversion of C-interrupted T homopolymers (i.e., TTTTCTTTTT) into long T or uracil interrupted homopolymers that subsequently acquire T deletions via strand slippage during replication (*SI Appendix*, Fig. S4*C*), the rate of which is reportedly >100-fold higher for A/T homopolymers of 10 vs. 4 nucleotides (50).

Consistent with our reporter data, the mean frequency of insT-ApoBPS per genome decreased in strains lacking Ung1 and increased in Mph1-deficient yeast (Fig. 3). Of the 243 complex insertion-SNV events, 86.4% were phased, 0.4% unphased, and 13.2% ambiguous, indicating the T insertion and APOBEC target BPS are linked events occurring on the same chromosome, as independent mutations would have a similar frequency of phased and unphased events (Datasets S3 and S4). These findings further support our proposed mechanism for insT-ApoBPS (Fig. 2*H*). In addition to insT-ApoBPS, 25 of 175 phased 1 bp- insertion-ApoBPS events occurred in disconnected homopolymers (*SI Appendix*, Fig. S7 and Datasets S3 and S4), suggesting these events also occur by TLS slippage events during abasic site bypass.

### Mechanisms Producing APOBEC-Induced DNVs and SNV-SNV Mutations.

**Fig. 4.**
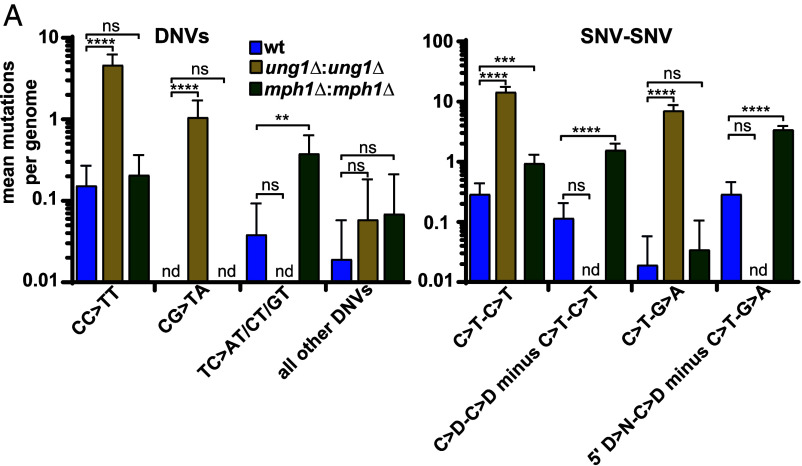
Subclasses of APOBEC-induced DNVs and *didyma*-like SNV pairs are differentially dependent on dU templating or TLS. (*A*) The mean frequency of phased dinucleotide variants (DNVs) events and paired closely spaced single nucleotide variants (i.e., *didyma*-like events) in WT, *ung1*Δ/*ung1*Δ, and *mph1*Δ/*mph1*Δ yeast expressing A3A. Numbers of sequenced genomes for each genotype is as in Fig. 3. Error bars indicate 95% CI. ***P < 0.001 and ****P < 0.0001 based on pairwise Mann–Whitney *U* tests. Not Significant (ns). Not Detected (nd). Nucleotide abbreviations follow the IUPAC nucleotide code (i.e., D = A, T, or G; N = any base).

In our yeast WGS data, the three most prominent DNV classes occurred primarily at T**CC**, T**CG**A, or **TC** sequences (APOBEC-target C is underlined, mutated dinucleotides bold font) and the frequency of each was heavily influenced by the absence of Ung1 or Mph1 (Figs. 3 and 4 and Dataset S4), suggesting both dU templating and bypass of abasic sites can elevate DNVs. For 82% of DNVs analyzed, the paired substitutions in each DNV event are phased (*P* = 2.2 × 10^−16^ by goodness of fit g-test), which indicates they occurred through a single mutagenic event. The only unphased DNVs were observed in Ung1 deficient yeast; of these unphased events, 17 of 20 were CG>TA DNVs. COSMIC doublet base substitution (DBS) signature 11, which is primarily composed of CC>TT mutations, has been associated with APOBEC signatures SBS2 and SBS13 (21). We found that the frequency of phased CC>TT mutations increased 34-fold in *ung1Δ*/*ung1Δ* yeast over WT and were unaffected by loss of Mph1 (Fig. 4), which indicates they are abasic site-independent. Because 89.4% of CC>TT mutations were phased (vs. 0.4% unphased) and their occurrence increases in the absence of Ung1, we propose these events are dU templated and form through processive deamination events in which deamination of the first cytidine produces a new APOBEC target motif UC, or TC (following DNA replication), that promotes subsequent deamination of the second cytidine (*SI Appendix*, Fig. S8*A*). CG>TA DNVs were also elevated in *ung1Δ*/*ung1Δ* yeast with 30 of 38 CG>TA DNV in TCGA contexts. Although CG>TA could occur in sequential, but linked events separated by a round of replication (i.e., converting TCGA to TCAA, a more favorable A3A/B target motif), approximately 46% of CG>TA DNVs are unphased, which indicates most of these DNVs are generated by two independent events. In addition to CC>TT mutations, TC>AT/CT/GT DNVs are a minor, but noticeable component of COSMIC DBS11 (*SI Appendix*, Fig. S9). We found that TC>AT/CT/GT DNVs were 100% phased (13/13 mutations), Ung1-dependent, and significantly increased in yeast lacking Mph1 (Fig. 4 and *SI Appendix*, Fig. S9). These results indicate that TC>AT/CT/GT DNVs occur during TLS bypass of APOBEC/Ung1-induced abasic sites where the TLS polymerase inserts an A opposite the abasic site and subsequently misincorporates across from the undamaged 5′ thymidine (*SI Appendix*, Fig. S8*B*). Less frequent TC>AG/CG/GG mutations seen in DBS11 might also occur via a similar mechanism, but we did not observe this class of DNVs in yeast.

*Didyma*, APOBEC-induced BPS co-occurring within small stretches of DNA (i.e., 3 to 50 nucleotides), may encompass diverse types of mutations formed by different mechanisms (25, 33). We classified SNV pairs that are within 10 bp of one another and occur on the same chromosome (phased in WGS reads) into four prominent groupings (Fig. 4). SNV pairs with an APOBEC C>D mutation at the 3′ position in the pair, C>D-C>D (excluding C>T-C>T), and directional 5′ D>N-C>D (excluding C>T-G>A) were Ung1-dependent and significantly increased in strains lacking Mph1 (Fig. 4), which indicates that these events primarily occur during lesion bypass of A3A and Ung1 generated abasic sites. C>T-C>T and C>T-G>A SNV pairs were increased in *ung1Δ*/*ung1Δ* yeast; however, only 49% and 53% of the observed DNVs were phased, respectively, which indicates that most of the BPS in SNV-SNV pairs occurred independently. Conversely, 88% and 100% of C>T-C>T SNV-SNV pairs were phased in WT and Mph1 deficient yeast, respectively. The frequency of C>T-C>T SNV pairs were increased in yeast lacking Mph1 (Fig. 4), which indicates the BPSs in these SNV-pairs occur due to bypass of abasic sites by TLS in cells with functional UNG. For phased SNV pairs in WT and Mph1 deficient yeast, we assessed if these events were intergenic, or located on the transcribed strand (TS) or nontranscribed strand (NTS) and found that the frequencies of events did not differ (chi-square goodness of fit, *P* = 0.473) from those predicted by frequency of TC motifs in intergenic regions and on the TS and NTS strands of genes (Dataset S5), which indicates these events are not associated with transcription.

### Noncanonical APOBEC-Induced Mutations Cultured in Human Cells.

**Fig. 5.**
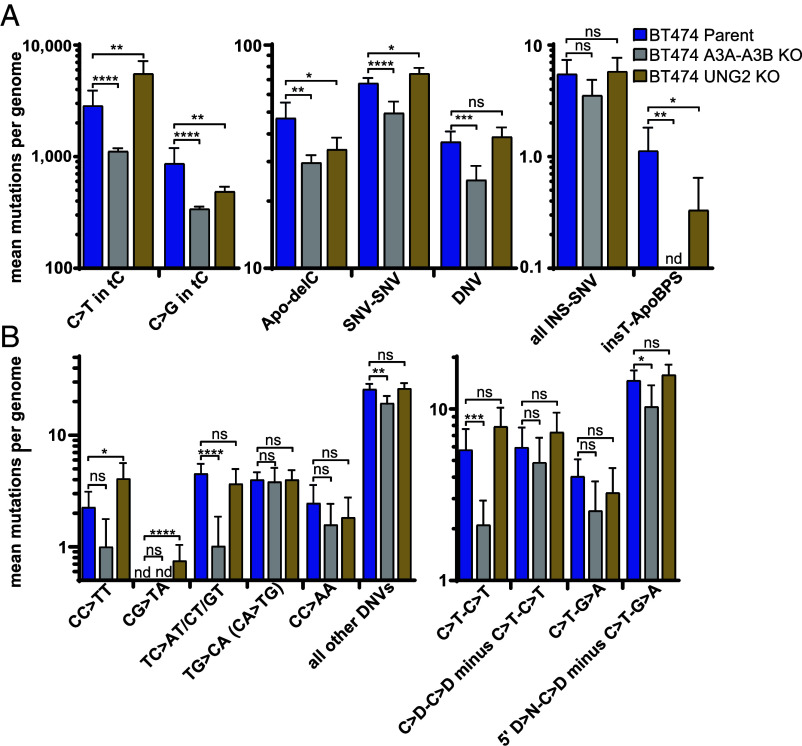
Dependence of noncanonical APOBEC-induced mutations on APOBEC3A/B and UNG2 in BT474 cells. (*A*) Mean number of canonical APOBEC signature substitutions (C to T in tC and C to G in tC) and noncanonical APOBEC-induced mutations (Apo-DelC, *didyma*-like SNV-SNV, DNV, INS-SNV, and insT-ApoBPS) per genome in parental, A3A/A3B double KO, and UNG1 KO BT474 outgrowth lines. (*B*) Mean number of events for different classes of DNV and *didyma*-like mutations in parental, A3A/A3B double KO, and UNG1 KO BT474 outgrowth lines. 15, 9, and 12 independent genomes were analyzed for parental, A3A/A3B double KO, and UNG1 KO BT474, respectively. *P < 0.05, **P < 0.01 ***P < 0.001 and ****P < 0.0001 based on pairwise Mann–Whitney *U* tests. Not Significant (ns). Not Detected (nd). Nucleotide abbreviations follow the IUPAC nucleotide code (i.e., D = A, T, or G; N = any base).

To determine if endogenous levels of A3A/A3B are sufficient to produce noncanonical APOBEC-induced mutations in human cells, we analyzed WGS of clonal outgrowths of the breast cancer cell line BT474, which are known to accumulate A3A-dependent SBS2/13 mutations in culture, and BT474 derived knockout cell lines (20). This reanalysis confirmed that C>T and C>G substitutions at TC dinucleotide were both reduced in the A3A/A3B knockout lines while UNG2 knockout resulted in reciprocal changes in these substitution types (i.e., increased C>T, decreased C>G; Fig. 5*A* and Datasets S6–S10). Consistent with our results from yeast, the Apo-delC mutations were significantly reduced in the A3A/A3B, and UNG2 knockout clones (Fig. 5*A* and Datasets S6–S10). Moreover, these mutations predominantly occurred at TC dinucleotides flanked by a 5′ polyT tract (*SI Appendix*, Fig. S10). We also found that the fraction of Apo-delC mutations that disrupt long homopolymers was increased in BT474 cells with a knockout of SMUG1, a uracil DNA glycosylase that prefers dsDNA and likely acts outside of DNA replication (51) (*SI Appendix*, Fig. S11), which indicates that the dU templating mechanism (*SI Appendix*, Fig. S4*C*) can generate Apo-delC mutations in human cells. In addition, the insT-ApoBPS events that occurred in BT474 cells were also decreased in A3A/A3B and UNG2 deletion lines (Fig. 5*A* and Datasets S6–S10). Together these findings indicate that endogenously expressed A3A/A3B produce both Apo-delC and insT-ApoBPS mutations primarily through the generation of abasic sites adjacent to neighboring homopolymer runs enabling primer and template slippage during TLS bypass. Apo-delC and insT-ApoBPS mutations appear similarly dependent on A3A/A3B expression in MB-MDA-453 breast cancer cells (*SI Appendix*, Fig. S12), though the small number of sequenced samples makes the insT-ApoBPS comparison statistically insignificant.

Approximately 97% of DNVs in BT474 and BT474 derived cell lines were phased (the remaining 3% of DNVs were ambiguous), indicating that they occur by singular events that generate two adjacent BPS (Dataset S10). CC>TT DNVs, the primary component of DBS11, were increased in the UNG2 KO lines compared to parental BT474 (Fig. 5*B*), which, like our findings in yeast, indicates that these events are generated by subsequent deamination events and dU templating (*SI Appendix*, Fig. S8*A*). TC>AT/CT/GT DNVs, a minor component of DBS11, were clearly reduced in BT474 cells lacking A3A/A3B but were unaffected by loss of UNG2. All SNV-SNV pairs were reduced in BT474 A3A/A3B KO lines, but only C>T-C>T and D>N-C>D (excluding C>T-G>A) pairs reached statistical significance compared to the parental line (Fig. 5*B*).

### Noncanonical APOBEC-Induced Mutations in Primary Tumors.

**Fig. 6.**
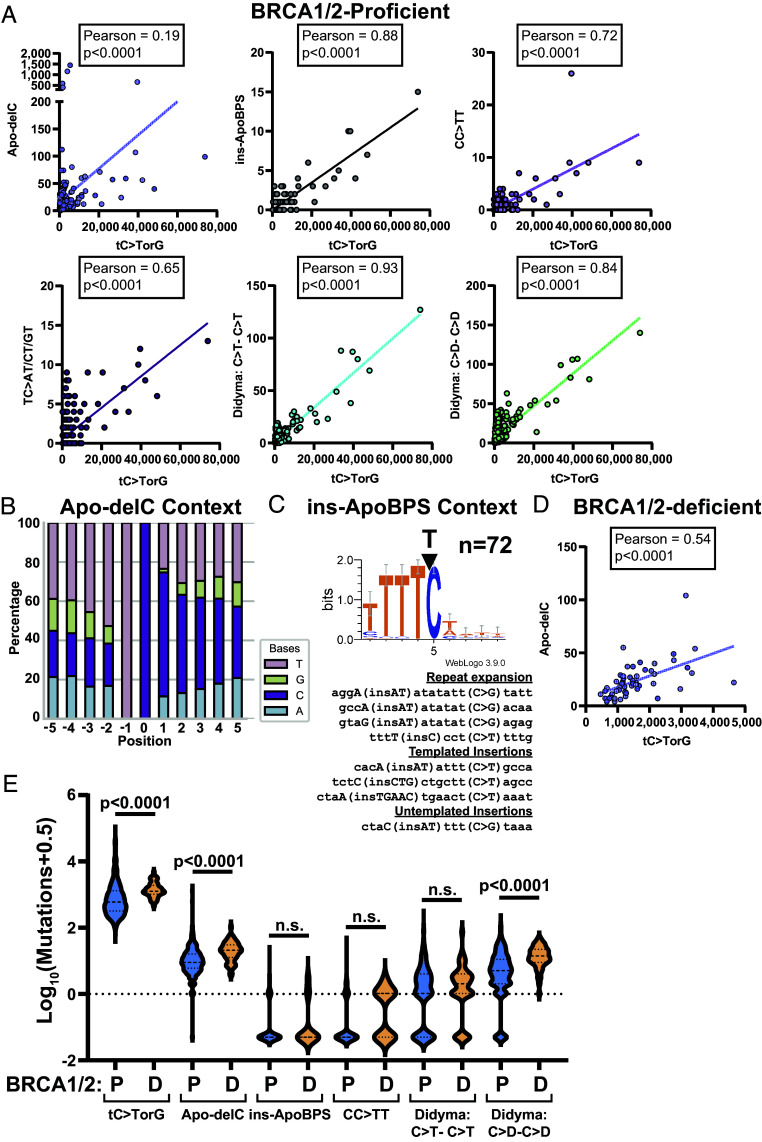
Noncanonical APOBEC-induced mutations in primary breast cancers. (*A*) Correlation of Apo-DelC, Ins-ApoBPS, CC>TT, TC>AT/CT/GT, C>T-C>T *didyma*, and C>D-C>D (excluding C>T-C>T) *didyma* events with canonical APOBEC-induced substitutions (i.e., C to T or G in tC) in 515 BRCA1/2-proficient breast cancers. *P*-values and correlation coefficients are calculated using a Pearson correlation analysis. (*B*) ±5 bp context of Apo-DelC mutations showing enrichment of T bases 5′ of the deleted C. (*C*) Weblogo (53) of ±5 bp context of insT-ApoBPS mutations in BRCA1/2-proficient breast cancers. Contexts are centered on the ApoBPS mutation. 72 events are included. Location of inserted T bases is indicated. Insertions within 10 nucleotides 5′ of an ApoBPS mutation that diverge from the insT-ApoBPS model are shown categorized by insertion type. (*D*) Correlation of Apo-DelC with canonical APOBEC-induced substitutions (i.e., C to T or G in tC) in 54 BRCA1/2-deficient breast cancers. *P*-values and correlation coefficients were generated as in (*A*). (*E*) Impact of BRCA1/2 deficiency on putative noncanonical APOBEC-induced mutations. 515 and 54 BRCA1/2-proficiency and -deficient tumors were analyzed. *P*-values were determined by the Mann–Whitney *U* test. Not Significant (ns). Nucleotide abbreviations follow the IUPAC nucleotide code (i.e., D = A, T, or G; N = any base).

We next assessed the presence of noncanonical APOBEC-induced mutations in 569 whole genome sequenced breast cancers (52). 54 tumors in this cohort carried germline mutations in either *BRCA1* or *BRCA2*, leaving the remaining 515 tumors proficient in HR. Within the BRCA1/2-proficient tumors, Apo-delC, ins-ApoBPS, CC>TT, TC>AT/CT/GT, *didyma*-like C>T-C>T, and C>D-C>D mutation types significantly correlated with the number of canonical APOBEC-induced substitutions (Fig. 6*A* and Datasets S11 and S12), suggesting that all these mutation types are increased by APOBEC activity. Ins-ApoBPS, CC>TT, *didyma*-like C>T-C>T, and C>D-C>D events correlated very strongly, displaying Pearson correlation coefficients greater than 0.7. The correlation of Apo-delC mutations in BRCA1/2 proficient tumors (Pearson = 0.19; *P* < 0.0001), is weakened by nine tumors with high SBS6 (i.e., mismatch repair deficient) that have extremely high numbers of C deletions in C homopolymers and are not enriched for APOBEC SBS signatures. In BRCA1/2-deficient tumors, the majority of which have moderate levels APOBEC-induced BPS, Apo-delC mutations correlated more strongly with SBS2/13 mutations (Fig. 6*D*). Analysis of the surrounding sequence context indicated that the deleted cytidines occurred with neighboring 5′ polyT sequences (Fig. 6*B*) like the Apo-delC mutations observed in yeast and breast cancer cell lines, and thus likely occur by a similar mechanism.

Ins-ApoBPS mutations in breast cancers also displayed similar characteristics to those described in yeast and breast cancer cell lines. Most of these complex events (n = 72) involved C>T or C>G substitutions with 5′ T insertions within a polyT tracts (Fig. 6*C*), mirroring the APOBEC-induced mutations observed in yeast and human cell lines. However, other Ins-ApoBPS mutations were observed that could be classified into three additional groups:those with repeat expansions where the inserted bases represent an additional unit of a neighboring repeat, similar to the insCT-ApoBPS we observed in yeast (*SI Appendix*, Fig. S2*C*) that were Ung1 and Rev1/Rev3 dependent (Datasets S1 and S2), templated insertions that create direct repeats, and untemplated insertions.

We previously showed that the genomes of BRCA1/2-deficient breast cancers contain higher amounts of APOBEC-induced SBS mutations, which was driven by increased SBS13 mutations (31), suggesting that these proteins function to facilitate HR-dependent template switching to bypass APOBEC-induced abasic sites in the context of DNA replication similar to mechanisms established in yeast (34) and DT40 cells (37, 38). Comparison of APOBEC-induced mutation frequencies in BRCA1/2-proficient and deficient breast cancers recapitulated these prior analyses for canonical APOBEC-induced substitutions (Fig. 6*E*). As observed in *mph1*Δ yeast, Apo-delC and C>D-C>D *didyma*-like events were also significantly elevated in BRCA1/2 deficient tumors, while HR deficiency had minor impacts on CC>TT and C>T-C>T *didyma* events. Unlike our yeast data, HR deficiency did not elevate the INS-ApoBPS mutations. However, BRCA1/2 deficient tumors are enriched in A3B-like (RTCA preferred motif) mutations as opposed to A3A-like (YTCA preferred motif) mutations (31). Because insT-ApoBPS mutations occur almost exclusively in TTC (YTC) motifs, reduced deamination at TTC motifs in A3B-mutated, BRCA deficient tumors compared to A3A-mutated BRCA1/2 proficient tumors could explain this discrepancy. Additionally, insT-ApoBPS are the rarest noncanonical APOBEC-induced mutations in human cells and the most likely to be under called in tumor WGS data, which makes such comparisons difficult.

## Discussion

Using both a yeast model system and analysis of mutations from breast cancer cell lines, we conclusively demonstrated that A3A and A3B cause Apo-delC and insT-ApoBPS mutations. In addition, these mutations correlate with APOBEC SBS signature mutations in breast cancer tumors (Fig. 6). We presented multiple corroborating data (Figs. 2, 3, and 5) indicating that insT-ApoBPS mutations happen during TLS bypass of Ung1/UNG2-dependent abasic sites initiated by A3A and A3B activity (Fig. 2 *D* and *H*). In the case of Apo-delC mutations, we provided evidence supporting three separate mechanisms that can generate these mutations in different contexts (Fig. 2*D* and *SI Appendix*, Fig S4). A decrease in Apo-delC events in BT474 UNG2 KO cell lines (Fig. 4), sequence context (Fig. 6 and *SI Appendix*, Fig. S10), and enrichment of these events in BRCA1/2 deficient tumors (Fig. 6) all support an A-rule insertionTLS extensiontemplate slippageextension model (Fig. 2*D*) as the primary mode that generates these events in APOBEC-mutated breast cancer tumors. Another study that reclassified indel signatures by considering flanking sequences, also found that C deletions in TTC, which they termed InD9a, were associated with APOBEC SBS signatures and were enriched in a large fraction of both lung and bladder cancer tumors (54), which are often APOBEC-mutated (55). Consequently, the authors also found two additional C deletion signatures without a preceding T homopolymer, InD9b and InD9c (54), which could occur by the A-rule insertionslippageextension model we proposed for C deletions in Rev1 and Rev3 deficient and *pol3-01* yeast (*SI Appendix*, Fig. S4). These findings support earlier studies (56, 57) that show Polδ can bypass abasic sites without the aid of specialized TLS polymerases in some contexts. From WGS data of Ung1 deficient yeast expressing A3A and BT474 cells lacking SMUG1 (Fig. 3 and *SI Appendix*, Figs. S6*B* and S11), we found evidence supporting a separate mechanism for C deletions occurring in long C-interrupted T homopolymers (*SI Appendix*, Fig. S4*C*).

In yeast, Apo-delC and insT-ApoBPS had similar rates whether measured by reporters (Fig. 2) or in WGS data (Fig. 3). However, in cultured human cells Apo-delC mutations are approximately 42-fold more frequent than insT-ApoBPS mutations. Based on our finding that insT-ApoBPS mutations are driven primarily by Rev1 insertion of C opposite abasic sites (Fig. 2), insT-ApoBPS should be the more frequent of these two mutation classes in BT474 cells where C>G mutation make up approximately 25% of the APOBEC SBSs, while in yeast C>G mutations represent only 2% of APOBEC SBSs in WGS data. Mutations in both these datasets were similarly called with Strelka2, so a difference in mutation calling is unlikely to account for this discrepancy. We posit that mechanistic differences in TLS reduce primer strand slippage events during bypass of abasic sites in human cells. Although there are differences between yeast and human TLS (i.e., additional human TLS polymerases and minor differences in hREV1 and hPolζ structure and functions), none of these differences offer an apparent explanation for the scarcity of insT-ApoBPS mutations in human cells.

Our data indicate that CC>TT mutations and TC>AT/CT/GT mutations, which comprise DBS11, occur by uracil templated synthesis and TLS-dependent bypass of APOBEC/UNG-dependent abasic sites, respectively (Figs. 4 and 5). In our defined yeast model for APOBEC-induced mutagenesis, we found that A3A/A3B-induced paired SNVs C>T-C>T, C>D-C>D (excluding C>T-C>T) and 5′ D>N-C>D 3′ (excluding C>T-G>A) are elevated in Mph1 deficient yeast that lack abasic sites bypass by HR-dependent template switching, which indicated these mutations occur due to TLS synthesis. In addition to our model for *didyma* mutations, others have shown that deamination of hairpin loops (25) and repair of lesions during transcription (33) can generate APOBEC *didyma* mutations. Consequently, the relative contribution of these mechanisms to *didyma* mutations in different cell contexts and the roles these mutations have in cancer biology is still unclear.

In addition to these complex SNV-SNV mutations, we noticed that many of the insT-ApoBPS events in yeast had an additional C>T or C>G mutation further 5′ the 5′ T homopolymer (*SI Appendix*, Fig. S3), which may indicate TLS bypass events may increase nearby APOBEC signature BPSs. It is likely that multiple polymerase switches and other activities required for TLS bypass are significantly slower than normal templated synthesis, which may increase the duration of ssDNA 5′ of APOBEC-induced abasic sites at effected Okazaki fragments, thereby locally increasing mutations caused by APOBEC deamination. Although our analysis indicates that similar mechanisms promote these APOBEC-induced non-SBS mutations in human cells, further work is needed to fully understand the repair processes that modulate these mutations. For example, unlike in our yeast model, APOBEC-induced SNV-SNV pairs and TC>AT/CT/GT DNVs were not reduced in UNG2 deficient BT474 cells, which may be due to functional redundancy between SMUG1 and UNG2 in human cells (20) for removal of APOBEC-generated dU.

Aside from demonstrating that APOBEC deaminases can cause mutations besides those defined by SBS2 and SBS13, the abasic site-dependent mechanisms supported by our data are likely applicable to other sources of abasic sites including spontaneous depurination, and repair of both endogenous and exogenous DNA damage, which are often enriched in tumor cells. Although abasic sites generated by glycosylases acting on random oxidized and alkylated bases throughout the genome are less likely to stall DNA synthesis than those generated by removal of A3A/A3B-induced dU, which is generated primarily on the lagging strand template during replication, they do generate abasic sites that are bypassed by TLS to some extent and may generate indels and complex mutations similar to those characterized in this study. Furthermore, mutations akin to insT-ApoBS were found to occur in yeast lacking Rad14 (58, 59), a homolog to human XPA, that is required for nucleotide excision repair, but with C>A and G>T mutations adjacent to T homopolymers. We hypothesize that similar mutations may be elevated in individuals with xeroderma pigmentosum caused by loss of functional XPA. We also found that the *pol3-01* mutations, which eliminated Polδ proofreading, significantly increase Apo-delC mutations (Fig. 2) and generated similar mutations in DTC motifs (*SI Appendix*, Fig. S2). Consequently, *POLE* and *POLD1* mutations that cause decreased exonucleolytic proofreading predispose carriers to colorectal and endometrial cancer and generate hypermutated, microsatellite-stable tumors (60). Hence, strand slippage during abasic site bypass might contribute to deletions in tumor cells with *POLE* and *POLD1* exonuclease mutations.

SBS2 and SBS13 mutations caused by deamination of cytidine by A3A and A3B are two of the most prevalent signatures in sequenced tumors (21). However, few driver mutations have been attributed to APOBEC signature mutations beyond oncogenic PIK3CA E542K and E545K mutations (18, 24). However humanized mouse models show that expression of these enzymes is cancer-promoting without obvious APOBEC signature mutations as drivers (26). The indel signatures we have attributed to A3A and A3B may contribute to multiple phenomena important to cancer biology. For example, APOBEC-induced indels could contribute to the reversion of pathogenic *BRCA1/2* mutations, which are the predominate cause of PARP inhibitor (PARPi) resistance (61). Consequently, Apo-delC mutations are enriched in BRCA1/2 deficient tumors (Fig. 6). Additionally, frameshift mutations and complex mutations are known to generate more potent neoantigens (42, 43). Hence, the APOBEC-induced indels and complex mutations defined in this study could help explain studies that show enhanced responses to immunotherapy in APOBEC-mutated tumors (62, 63). Furthermore, determining if noncanonical APOBEC-induced mutations contribute to driver mutations could aid in defining the role of APOBEC-induced mutations in tumorigenesis. We believe the work presented here will provide a framework for additional studies to characterize roles of APOBEC3-mutagenesis in cancer biology.

## Materials and Methods

### Yeast Strains and Growth Conditions.

Plasmids pTM-934 and pTM-935 (Dataset S13), which contain the sequence of the reversion reporters depicted in Fig. 2*A*, were PCR amplified with primers oTM-1227 and oTM-1170 that contain homology directing integration 522 base pairs upstream of the endogenous *CAN1* ORF. These PCR products were transformed into the BY4741 yeast strain generating strains yAP05 and yAP06, the parental, WT strains for measurement of 3z+1 and 3z−1 frameshift mutations (z = integers such as −3, −2, −1, 0, 1, 2…), respectively. Targeted integration was confirmed by PCR using oligos oBV-36 and oTM-1176. Yeast strains with gene deletions were made by transformations with PCR generated deletion cassettes and confirmed by PCR as in ref. 34. ySR128-derived yeast strains described in refs. 15, 34, 64, and 65 were used to measure *his7-2* reversion rates. CRISPR-Cas9 was used to generate *rev1-D467A, E468A* yeast strains similarly to (65). To avoid accumulation of secondary and/or suppressor mutations in yeast strains, strains were transformed with APOPBEC expression plasmids, pySR419 (vector control), pSR440 (A3B), or pVH-19 (A3A), immediately prior to measuring mutation rates and producing mutation spectra. The sequences of oligos and plasmids used to generate and validate yeast strains can be found in (Dataset S13). Descriptions and genotypes for all yeast strains can be found in Dataset S14.

### Mutation Rates.

For each reversion mutation rate measurement using yAP-05, yAP-06, and their derivatives, yeast strains transformed with APOBEC plasmids (Dataset S13) were grown from single cells on YPDA + Hygromycin + NTC agar plates to generate independent clonal cultures. Nine separate 10 ml cultures were grown in liquid YPDA + Hygromycin + NTC overnight to saturation and subsequently diluted and plated using volumes to yield between 30 and 200 colonies per plate on selective complete media (SC) lacking uracil, which selects for cells with reversion mutations, and nonselective SC media. Subsequently, colonies were counted, and the mutation rates were calculated using the Drake equation (66). For strains with deletions, two to three independent rates were calculated using independently generated isolates of each deletion strain to ensure reproducibility (Dataset S2). These data were combined for calculation of a single mutation rate using the Drake equation (66) (Dataset S2, under “Rates calculated from combined data”) and Fig. 2. Measurement of reversion mutation rates of *his7-2* reversion events were done similarly, except for using YPDA + Hygromycin media for the outgrowth plates and media and SC-His for selection of reversion mutations.

### Mutation Reporter Spectra.

Canavanine resistant and Ura or His prototrophs were clonally isolated from independent cultures by streaking to colonies on appropriate media, then individual colonies were used to make a 1x3cm patch on YPDA plates, from which genomic DNA was isolated. For characterizing events restoring *URA3* expression (Fig. 2), the purified genomic DNA was PCR amplified using primers oTM1230 and oTM1231, and the products were Sanger sequenced using primer oTM1231. Similarly, genomic DNA from independent *his7-2* reversion events were PCR amplified using primers oTM1174 and 1175 and the subsequent products sequenced with the same oligos. Mutations in *CAN1* were identified using PacBio sequencing of barcoded amplicons as in ref. 31. For oligo sequences and descriptions see (Dataset S13).

### Whole Genome Sequencing of Yeast Expressing APOBEC3A.

Clonal cultures of diploid yeast strains were passaged 25 times on YPDA + hygromycin agar plates with each passage composed of approximately 25 population doublings (from 1 to ~1.6 × 10^7^ cells) as described in ref. 67. Mutagenized clonal isolates were obtained by plating propagated cells on SC-His media to select for his7-2 reversion. Genomic DNA was isolated, libraries constructed, and sequencing performed on an Illumina Hiseq 4000 as in ref. 65.

### Whole Genome Sequencing Alignment.

150 bp Illumina sequencing reads were aligned to either the saCCer3 reference genome (for yeast) or hg19 (for human) with BWA-MEM2 mem (68, 69) using the **-M** option. The resulting SAM file was sorted by name (**sort -n**), processed to adjust mate information (**fixmate -m**), then sorted by position (**sort**) for removal of duplicate mutations (**markdup -r)**, all using Samtools (70).

### Mutation Calling.

Mutations were called using Strelka2 (71) with default parameters. For yeast, we compared cultured yeast from different genotypes to a previously sequenced WT yeast of the same background (15). To adjust for variations in sequencing depth, numerous WT colonies were merged to reach a total read depth similar to that of the newly sequenced colonies. For breast cancer cell lines, outgrowth clones were compared to sequencing of an originating starting clone. VCF files were filtered to include only mutations with a variant allele frequency (VAF) of 0.4 (for yeast) or 0.2 (for breast cancer cell lines) and a PASS status in the FILTER column. Mutations that were present in greater than 25% of yeast samples were eliminated to reduce the risk of false positives from preexisting mutations. VCF files were converted to BED format for subsequent analyses.

### Complex Mutations.

Mutations were categorized as part of complex events if they occurred within 10 bp of a neighboring mutation from the same genotype. Complex events were further classified based on the combination of mutation types (Substitution, Insertion, and Deletion). Given the convention of left-justifying INDELs in homopolymer runs, we also analyzed their positions using right justification for comparison with the 10 bp window. All other analyses used the left justification.

### Correction of Homopolymer INDEL Justification.

INDELs were analyzed in their left-justified representation. Because left justification within homopolymer sequence can shift positional attribution relative to strand of origin, APOBEC motif status was evaluated using a strand-consistent procedure. Variants corresponding to bottom-strand events (G deletions) were reverse complemented together with their local reference context, left-justified within a homopolymer if applicable, and then assessed for APOBEC sequence context. Motif assignment was based on the presence of a TCN trinucleotide surrounding the altered base after justification. This approach ensures equivalent context evaluation across strands and avoids loss of motif detection arising from normalization conventions applied to INDEL mutations.

### Mutation Phasing.

Read-supported phasing was used to evaluate whether nearby somatic events arose on the same DNA molecule. For events grouped within a complex, each variant was assessed relative to its adjacent partner, and for DNVs, both substituted positions were evaluated jointly. Reads spanning the relevant interval were examined to determine whether the alternative alleles occurred on the same sequencing read (phased) or on different reads (unphased). Co-occurrence on the same reads indicates that the variants are present on the same DNA molecule and are consistent with originating from a single lesion or mutational event, whereas separation across reads suggests independent origins.

For each event, the fractions of reads supporting co-occurrence or separation were calculated and used for classification. Events were labeled phased when co-occurrence predominated (yeast: phased fraction > 0.25 and unphased fraction < 0.05, and human: phased fraction > 0.10 and unphased fraction < 0.05), unphased when separation predominated (yeast: unphased fraction > 0.50 and phased fraction < 0.10, and human: unphased fraction > 0.20 and phased fraction < 0.05), and ambiguous otherwise. Thresholds were adjusted between human and yeast datasets to accommodate differences in ploidy, as well as variation in sequencing depth and alignment characteristics. Full algorithmic details are provided in the deposited code.

### Counting Mutations.

Somatic mutation spectra were summarized by counting single-nucleotide variants (SNVs), DNVs, insertions, and deletions, including biologically relevant subclasses such as cytosine-derived SNVs and single-base cytosine deletions. Variants occurring independently were tallied directly, whereas mutations grouped into multievent complexes were collapsed and counted once per complex event and categorized according to their component mutation types. Complex categories were assigned based on the presence of mutation classes within each complex irrespective of positional order. For example, any complex containing at least one SNV and one deletion was classified as an SNV–DEL complex, regardless of which event occurred first. Complex events containing paired SNVs were further classified based on their substitution patterns evaluated across both native and reverse-complement sequence orientations to ensure strand-consistent labeling. DNVs were categorized using canonical strand-agnostic representations, and context-aware labels reflecting relevant sequence features, like a TCN SNV, were assigned where applicable. Full implementation details and classification rules are provided in the deposited code.

### Analysis of APOBEC-Induced SNV-SNV Analysis.

Both yeast and human cell-line SNV/SNV transcriptional-strand asymmetry analyses were performed using the same custom Python workflow, with the appropriate reference FASTA and NCBI RefSeq GTF annotation supplied for each dataset. Mutation calls were filtered and restricted to SNVs in pyrimidine-normalized APOBEC contexts, defined as TCA, TCT, TCG, or TCC with C>A, C>G, or C>T substitutions. G>N mutations were converted into C-centered mutation space by reverse-complementing the sequence context and assigning the mutated cytosine to the opposite genomic strand. APOBEC SNVs were assigned as intergenic or to the transcribed strand (TS) or nontranscribed strand (NTS) using transcript/gene strand annotations from the GTF file. Only phased complex events were analyzed. Qualifying events were required to contain at least two phased strand coordinated APOBEC SNVs and a nonambiguous consensus TS/NTS assignment. Each qualifying phased SNV-SNV cluster was counted as a single event. The frequencies of SNV-SNV events in intergenic, TS, and NTS sequences for WT and *mph1*Δ yeast were compared to the total frequency of TC motifs in each sequence class (expected) by a chi-square goodness of fit.

## Supplementary Material

Appendix 01 (PDF)

Dataset S01 (XLSX)

Dataset S02 (XLSX)

Dataset S03 (XLSX)

Dataset S04 (XLSX)

Dataset S05 (XLSX)

Dataset S06 (XLSX)

Dataset S07 (XLSX)

Dataset S08 (XLSX)

Dataset S09 (XLSX)

Dataset S10 (XLSX)

Dataset S11 (XLSX)

Dataset S12 (XLSX)

Dataset S13 (XLSX)

Dataset S14 (XLSX)

## Data Availability

Fastq sequencing reads for WGS of clonal yeast outgrowth isolates are deposited under SRA accession number PRJNA1420684 (72). Illumina sequencing reads for WGS of A3A and A3B, and UNG2 knockouts in MDA-MB-453 and BT474 breast cancer lines were previously deposited under accession ERP137590 (73). Lists of identified mutations from reporter sequencing, yeast WGS, and breast cancer cell line WGS are provided in Datasets S1, S3, S6, S7, S8, and S9. Mutation lists for sequenced primary breast cancers were obtained from the International Cancer Genome Consortium Legacy 25 K repository under project BRCA-EU, Bucket: icgc25k-open (74). BRCA1/2-deficency was assessed based upon the presence of BRCA1 or BRCA2 germline variants in the tumor as indicated in Dataset S20 of ref. 52. Custom code for correcting homopolymer INDEL justification, determining phasing of complex and DNV mutations, and determining the abundance of specific mutation types was written in python3. Code generated for this study is publicly available at https://doi.org/10.5281/zenodo.20724527 (75).
