# Supplementary material for: Distinct repair processes produce APOBEC-induced deletions, tandem substitutions, and complex mutations in yeast and human cells
Source: Proc Natl Acad Sci U S A. 2026 Jul 28;123(31):e2609794123. doi: 10.1073/pnas.2609794123 (PMC13438585; doi:10.1073/pnas.2609794123)
Supplement: Supplementary file 1 [file pnas.2609794123.sapp.pdf]

Supporting Information for

Distinct repair processes produce APOBEC-induced deletions, tandem substitutions, and complex mutations in yeast and human cells.

Alexandra Puzon[1*], Atri Raval[1,2*], Cameron Cordero[2,*], Dan Schiefen[2], Piotr A. Mieczkowski[3], Steven A. Roberts[1,2,#], and Tony M. Mertz[1,2,*,#]

[1]School of Molecular Biosciences and Center for Reproductive Biology, Washington State University, Pullman WA 99164, [2]Department of Microbiology and Molecular Genetics, University of Vermont, Burlington, VT 05405, [3]Department of Genetics, Lineberger Comprehensive Cancer Center, University of North Carolina, Chapel Hill, NC 27599, USA, *These authors contributed equally, #Co-corresponding authors

Contact email: S.A.R. (srober23@med.uvm.edu) T.M.M (tony.mertz@med.uvm.edu)

This PDF file includes:

Figures S1 to S12
Legends for Datasets S1 to S14
SI References

Other supporting materials for this manuscript include the following:

Datasets S1 to S14

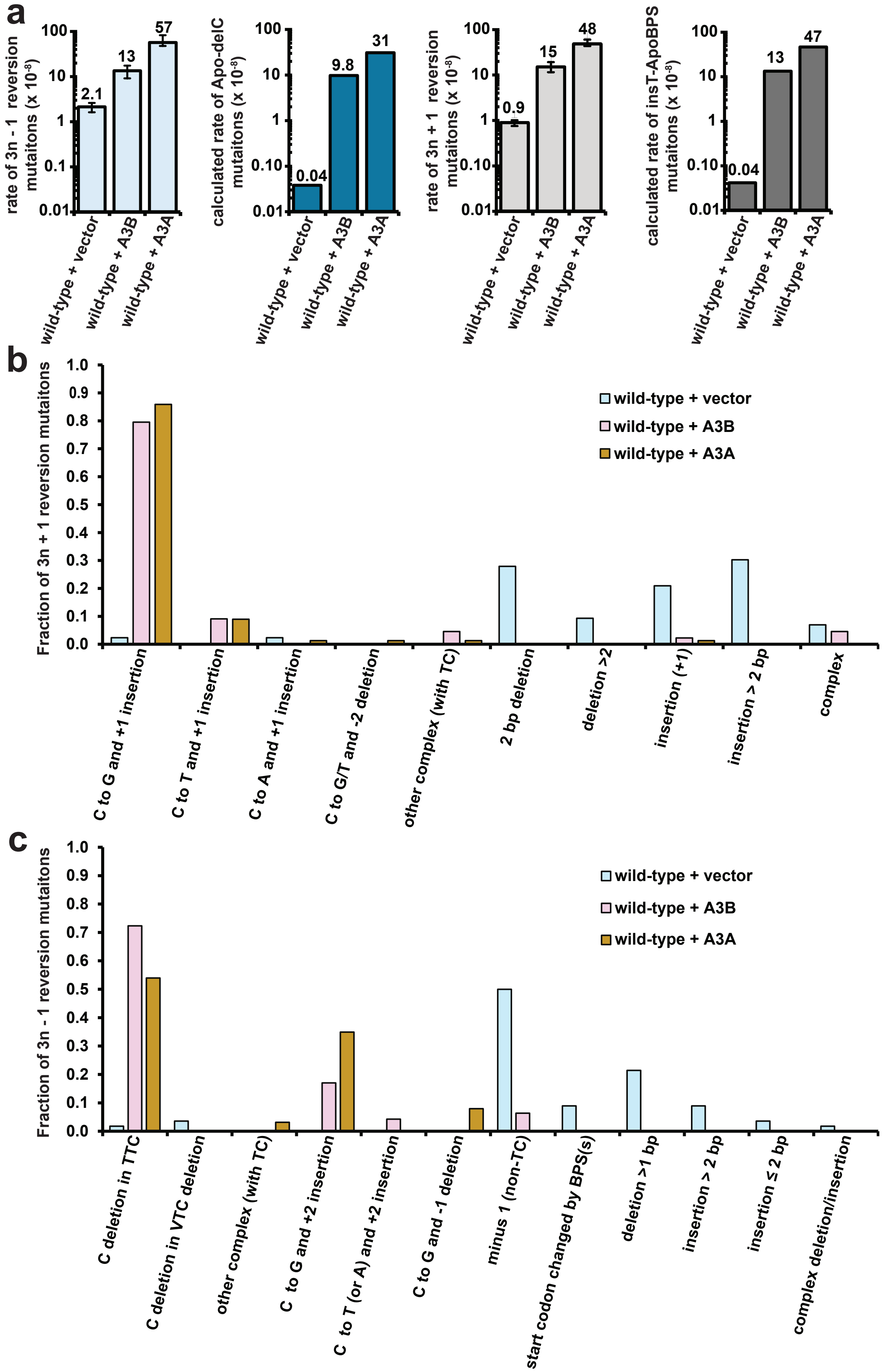

Supplementary Figure 1: Comparison of A3A and A3B reversion mutation rates and spectra. (a) Observed *URA3* reversion rates and calculated mutation rates. Error bars represent 95% confidence intervals and data labels display the numeric value of the mutation rate (×10^{-8}). **(b)** Spectra of 3n + 1 reversion mutations. **(c)** Spectra of 3n - 1 reversion mutations.

Supplementary Figure 2: The distribution of mutations mapped to the (3n-1) reversion reporter sequence. Note: This is a multi-page figure and the figure legend follows last panel.

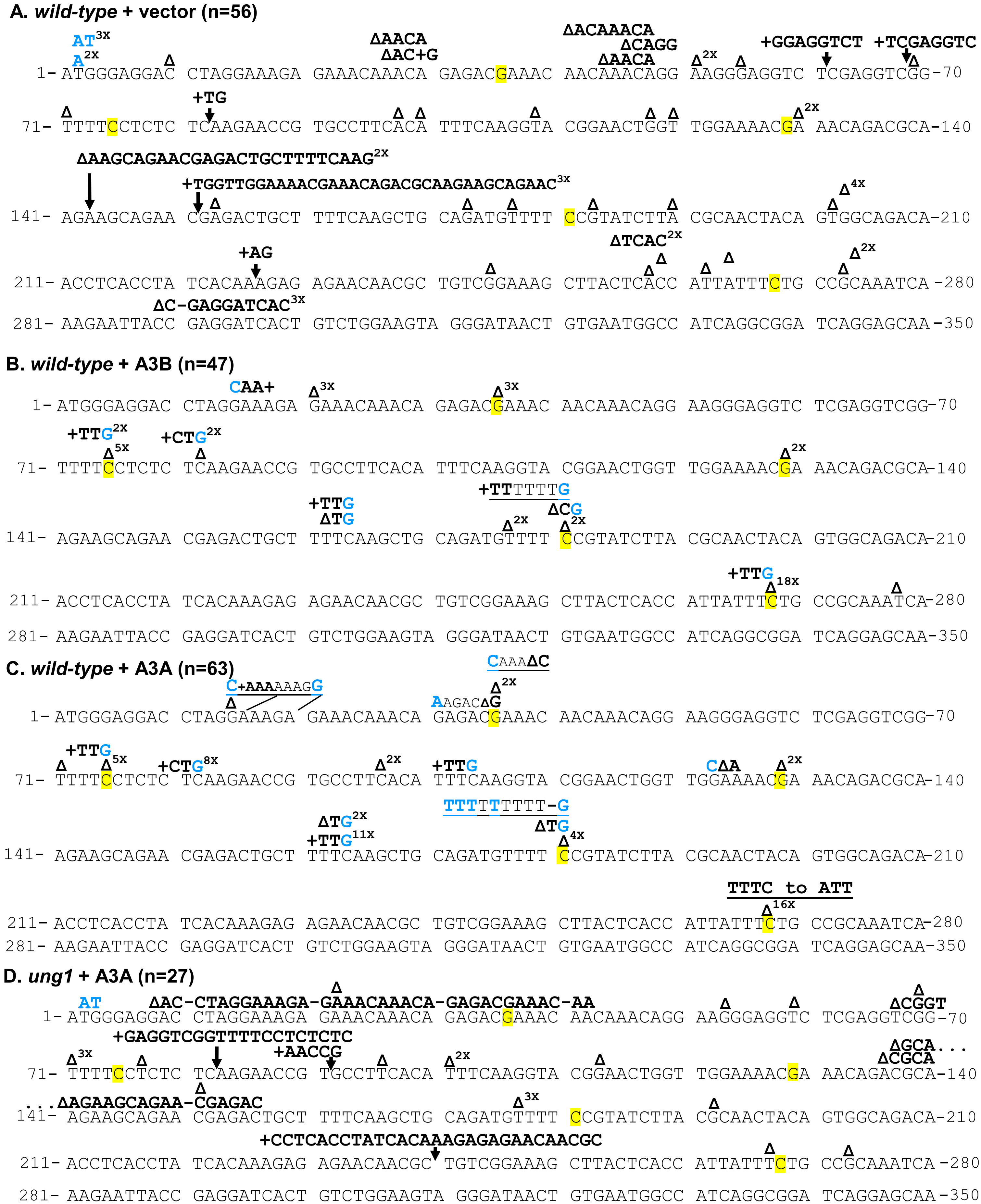

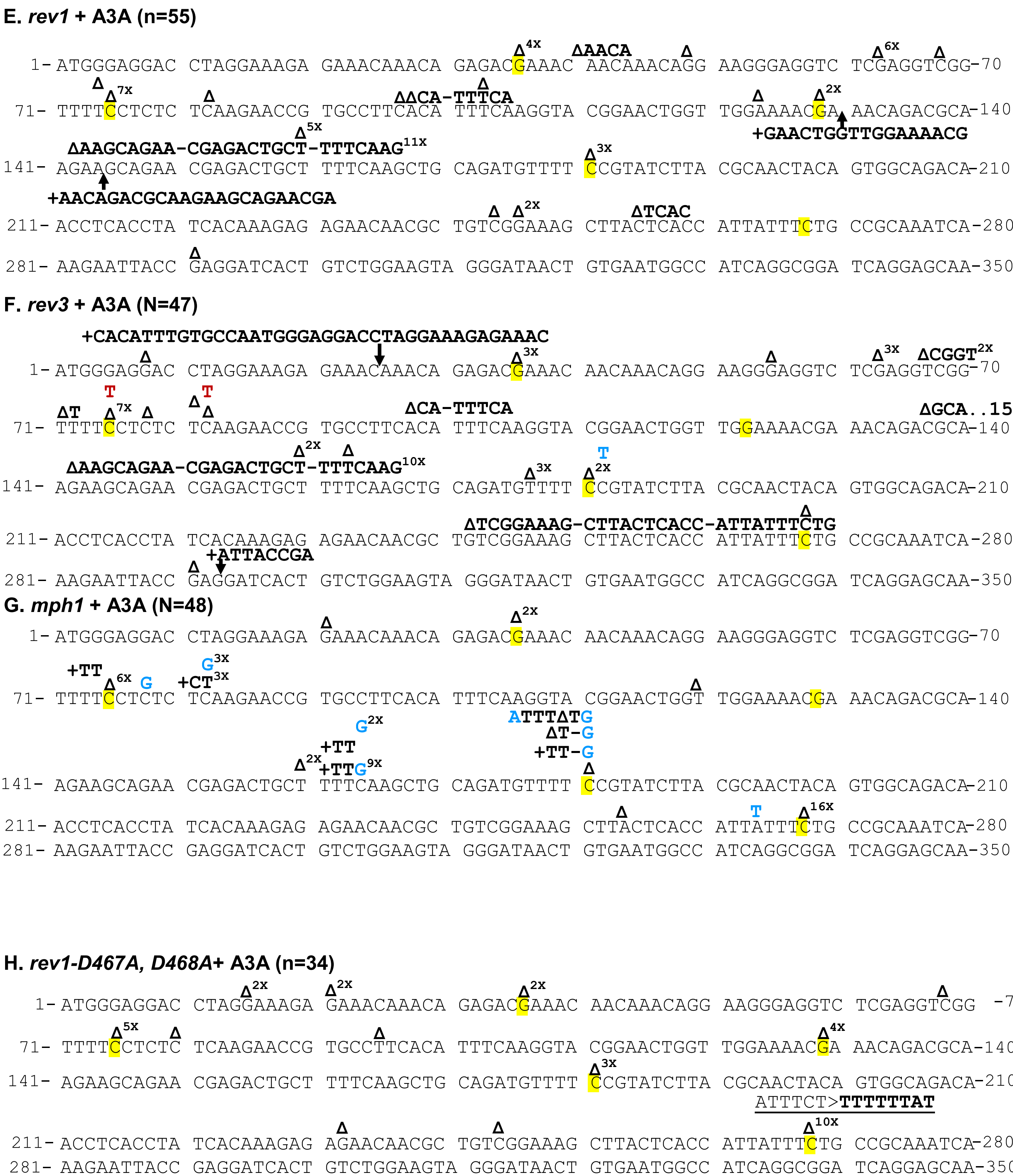
E. rev1 + A3A (n=55)
1- ATGGGAGGAC CTAGGAAAGA GAAACAAACA GAGACGAAAC AACAAACAGG AAGGGAGGTC TCGAGGTCGG-70
71- TTTTCCTCTC TCAAGAACCG TGCCTTCACA TTTCAAGGTA CGGAACTGGT TGGAAAACGA AACAGACGCA-140
141- AGAAGCAGAA CGAGACTGCT TTTCAAGCTG CAGATGTTTT CCGTATCTTA CGCAACTACA GTGGCAGACA-210
211- ACCTCACCTA TCACAAAGAG AGAACAACGC TGTCGGAAAG CTTACTCACC ATTATTTCTG CCGCAAATCA-280
281- AAGAATTACC GAGGATCACT GTCTGGAAGTA GGGATAACT GTGAATGGCC ATCAGGCGGA TCAGGAGCAA-350
ΔAACA
ΔΔCA-TTTCA
+GAACTGGTTGGAAAACG
ΔAAGCAGAA-CGAGACTGCT-TTTCAAG11x
+AACAGACGCAAGAAGCAGAACGA
ΔTCAC
F. rev3 + A3A (N=47)
1- ATGGGAGGAC CTAGGAAAGA GAAACAAACA GAGACGAAAC AACAAACAGG AAGGGAGGTC TCGAGGTCGG-70
71- TTTTCCTCTC TCAAGAACCG TGCCTTCACA TTTCAAGGTA CGGAACTGGT TGGAAAACGA AACAGACGCA-140
141- AGAAGCAGAA CGAGACTGCT TTTCAAGCTG CAGATGTTTT CCGTATCTTA CGCAACTACA GTGGCAGACA-210
211- ACCTCACCTA TCACAAAGAG AGAACAACGC TGTCGGAAAG CTTACTCACC ATTATTTCTG CCGCAAATCA-280
281- AAGAATTACC GAGGATCACT GTCTGGAAGTA GGGATAACT GTGAATGGCC ATCAGGCGGA TCAGGAGCAA-350
+CACATTTGTGCCAATGGGAGGACCTAGGAAAGAGAAAC
ΔCGGT2x
ΔT
ΔCA-TTTCA
ΔGCA..156
ΔAAGCAGAA-CGAGACTGCT-TTTCAAG10x
ΔTCGGAAAG-CTTACTCACC-ATTATTTCTG
+ATTACCGA
G. mph1 + A3A (N=48)
1- ATGGGAGGAC CTAGGAAAGA GAAACAAACA GAGACGAAAC AACAAACAGG AAGGGAGGTC TCGAGGTCGG-70
71- TTTTCCTCTC TCAAGAACCG TGCCTTCACA TTTCAAGGTA CGGAACTGGT TGGAAAACGA AACAGACGCA-140
141- AGAAGCAGAA CGAGACTGCT TTTCAAGCTG CAGATGTTTT CCGTATCTTA CGCAACTACA GTGGCAGACA-210
211- ACCTCACCTA TCACAAAGAG AGAACAACGC TGTCGGAAAG CTTACTCACC ATTATTTCTG CCGCAAATCA-280
281- AAGAATTACC GAGGATCACT GTCTGGAAGTA GGGATAACT GTGAATGGCC ATCAGGCGGA TCAGGAGCAA-350
+TT
+CT3x
+TTG9x
ATTTΔTG
ΔT-G
+TT-G
H. rev1-D467A, D468A+ A3A (n=34)
1- ATGGGAGGAC CTAGGAAAGA GAAACAAACA GAGACGAAAC AACAAACAGG AAGGGAGGTC TCGAGGTCGG -70
71- TTTTCCTCTC TCAAGAACCG TGCCTTCACA TTTCAAGGTA CGGAACTGGT TGGAAAACGA AACAGACGCA-140
141- AGAAGCAGAA CGAGACTGCT TTTCAAGCTG CAGATGTTTT CCGTATCTTA CGCAACTACA GTGGCAGACA-210
ATTTCT>TTTTTTAT
211- ACCTCACCTA TCACAAAGAG AGAACAACGC TGTCGGAAAG CTTACTCACC ATTATTTCTG CCGCAAATCA-280
281- AAGAATTACC GAGGATCACT GTCTGGAAGTA GGGATAACT GTGAATGGCC ATCAGGCGGA TCAGGAGCAA-350

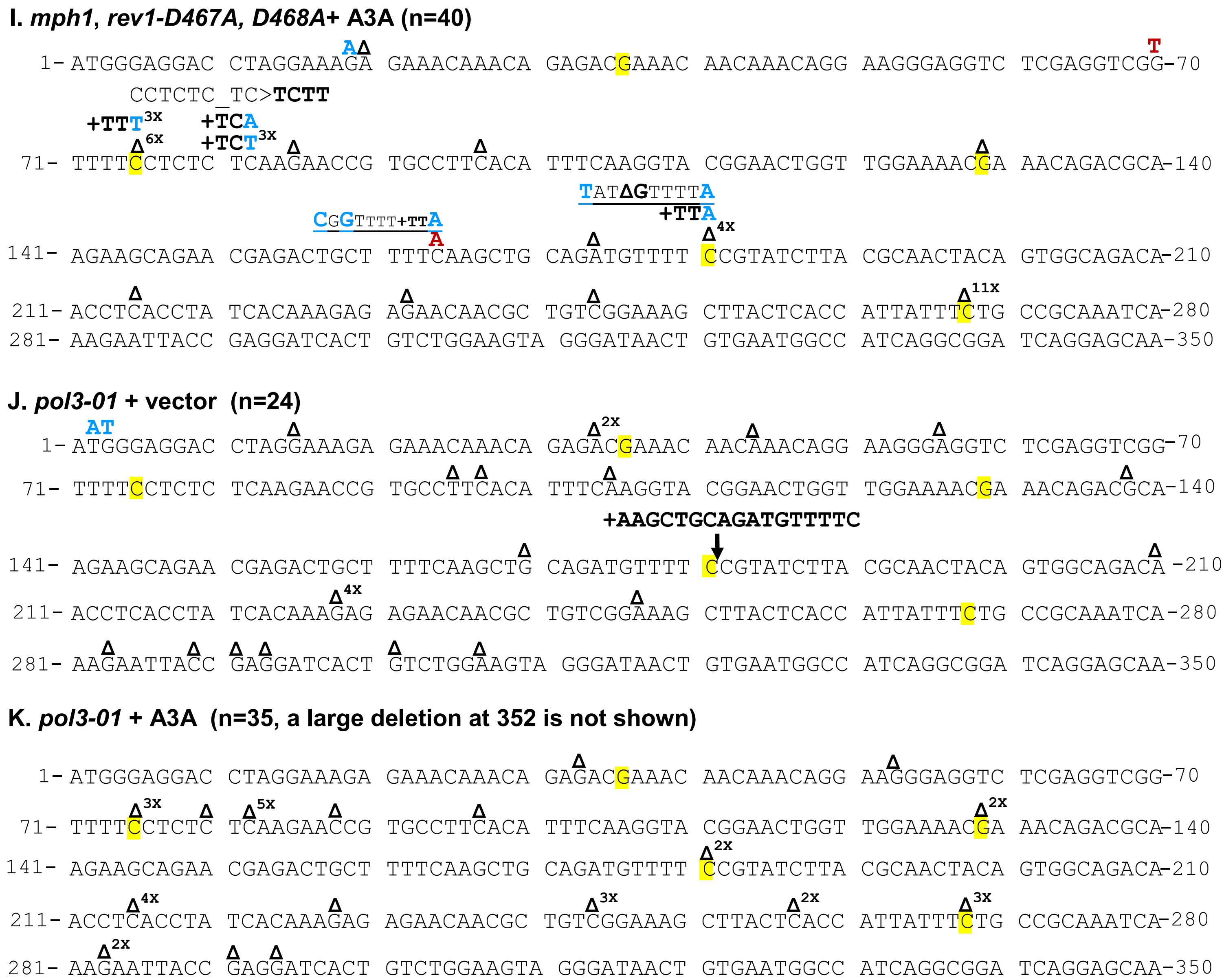

Supplementary Figure 2: The distribution of mutations mapped to the (3n-1) reversion reporter sequence. Mutations are in bold text. Delta (**Δ**) and plus (**+**) symbols indicated bases deleted and inserted bases, respectively. Base pair substitutions are in aqua blue font for simple and complex events. Base pair substitutions that are likely passenger mutations (distant from the indel events in the same sequenced sample) are in dark red font. Arrows indicate positions of large insertions. Unmutated bases within complex mutations are normal (not bold) font. Hotspots for Apo-delC mutations are highlighted yellow. (n) denotes the number of sequenced events. Underlining was used for complex events with multiple mutations separated by reference nucleotides to denote single mutation events. Superscripts denote multiples of the same mutation.

Supplementary Figure 3: The distribution of mutations mapped to the (3n+1) reversion reporter sequence. Note: This is a multi-page figure and the figure legend follows last panel.

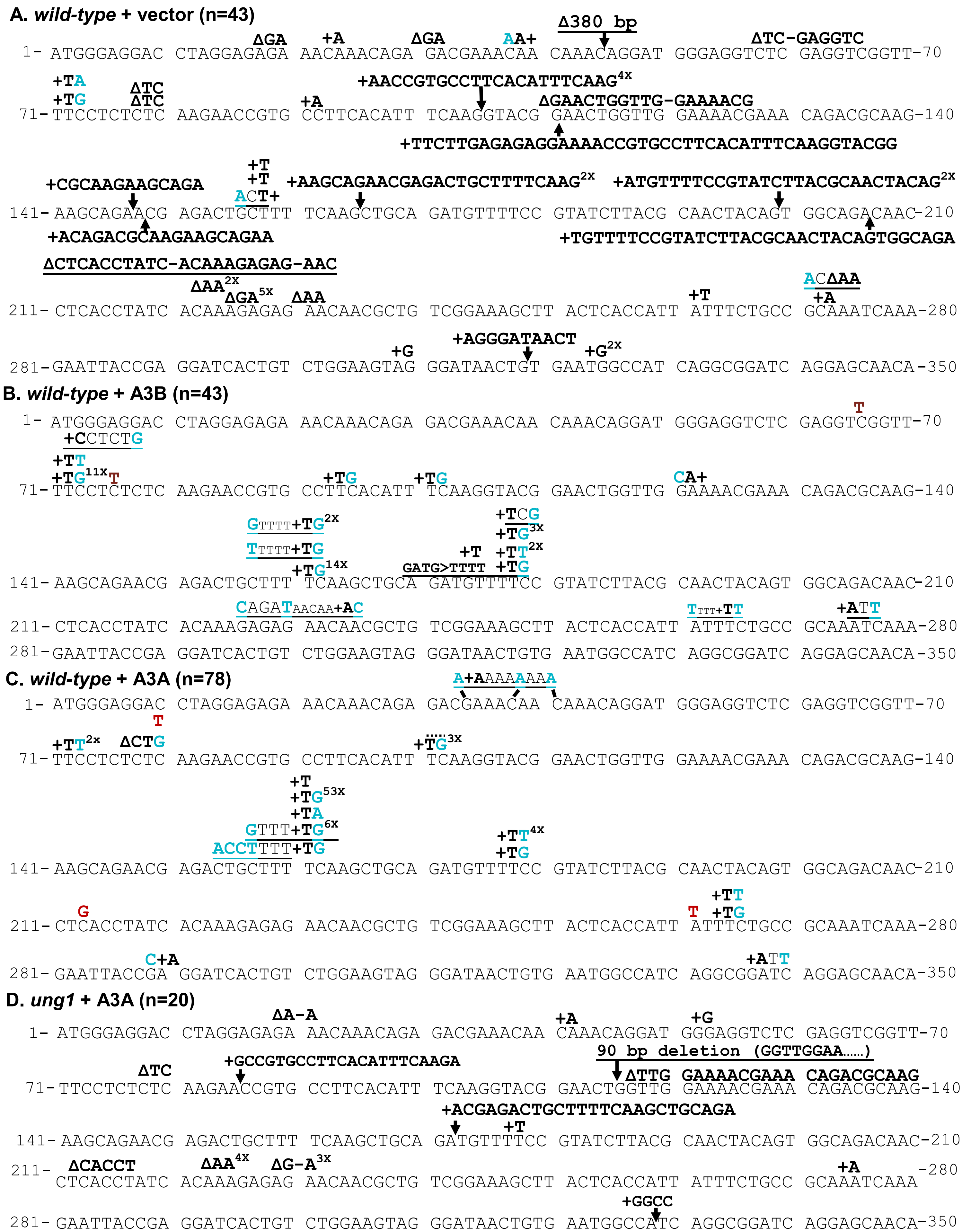

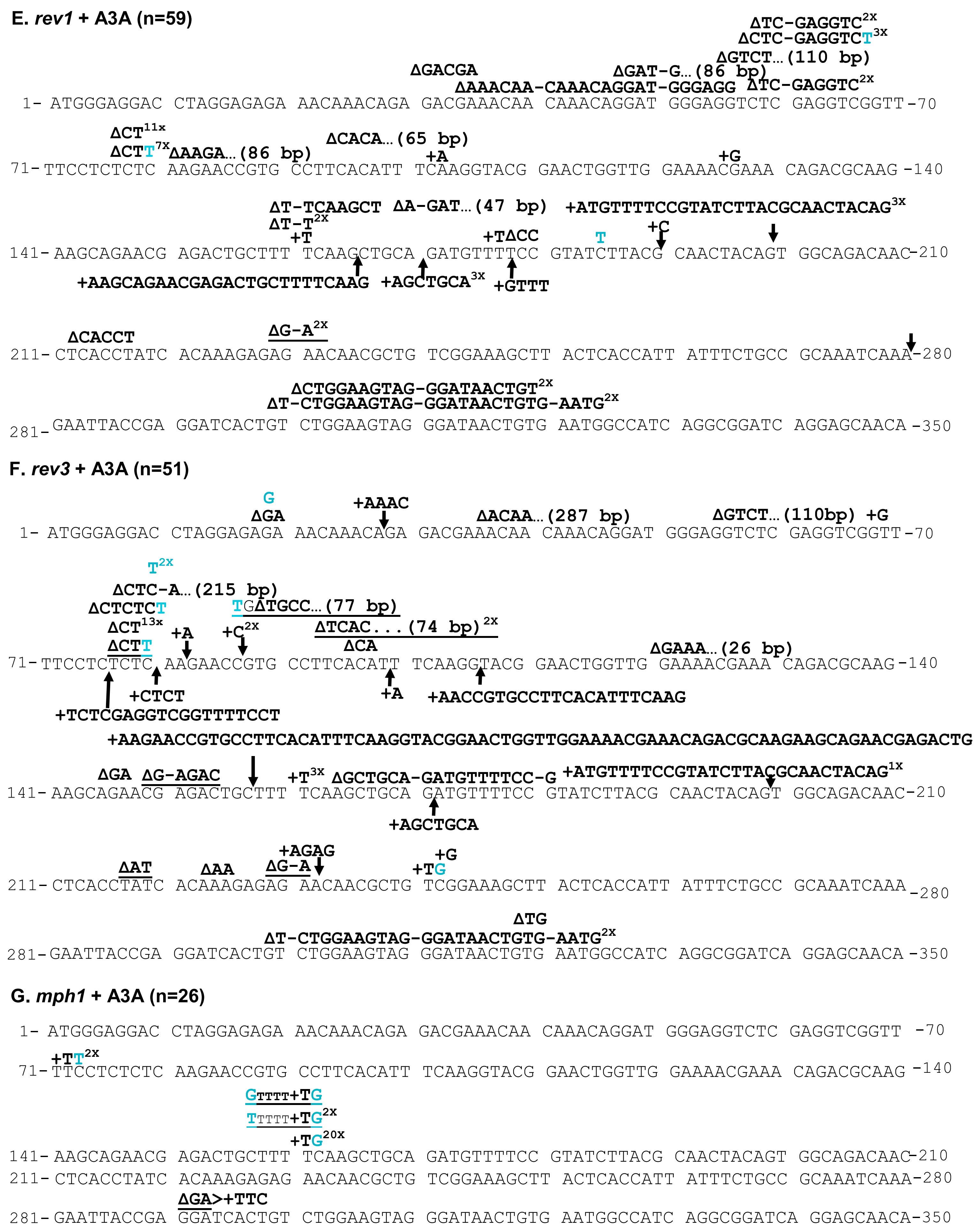

E. rev1 + A3A (n=59)
ΔTC-GAGGTC2x
ΔCTC-GAGGTCT3x
ΔGTCT... (110 bp)
ΔGACGA
ΔGAT-G... (86 bp)
ΔAAACAA-CAAACAGGAT-GGGAGG
ΔTC-GAGGTC2x
1- ATGGGAGGAC CTAGGAGAGA AACAAACAGA GACGAAACAA CAAACAGGAT GGGAGGTCTC GAGGTCGGTT -70
ΔCT11x
ΔCTT7x
ΔAAGA... (86 bp)
ΔCACA... (65 bp)
+A
+G
71- TTCCTCTCTC AAGAACCGTG CCTTCACATT TCAAGGTACG GAACTGGTTG GAAAACGAAA CAGACGCAAG -140
ΔT-TCAAGCT
ΔA-GAT... (47 bp)
+ATGTTTTCCGTATCTTACGCAACTACAG3x
ΔT-T2x
+T
+TΔCC
T
+C
141- AAGCAGAACG AGACTGCTTT TCAAGCTGCA GATGTTTTCC GTATCTTACG CAACTACAGT GGCAGACAAC -210
+AAGCAGAACGAGACTGCTTTTCAAG
+AGCTGCA3x
+GTTT
ΔCACCT
ΔG-A2x
211- CTCACCTATC ACAAAGAGAG AACAACGCTG TCGGAAAGCTT ACTCACCATT ATTTCTGCC GCAAATCAAA -280
ΔCTGGAAGTAG-GGATAACTGT2x
ΔT-CTGGAAGTAG-GGATAACTGTG-AATG2x
281- GAATTACCGA GGATCACTGT CTGGAAGTAG GGATAACTGTG AATGGCCATC AGGCGGATC AGGAGCAACA -350
F. rev3 + A3A (n=51)
G
ΔGA
+AAAC
ΔACAA... (287 bp)
ΔGTCT... (110bp)
+G
1- ATGGGAGGAC CTAGGAGAGA AACAAACAGA GACGAAACAA CAAACAGGAT GGGAGGTCTC GAGGTCGGTT -70
T2x
ΔCTC-A... (215 bp)
ΔCTCTCT
TGΔTGCC... (77 bp)
ΔCT13x
+A
+C2x
ΔTCAC... (74 bp)2x
ΔCTT
ΔCA
ΔGAAA... (26 bp)
71- TTCCTCTCTC AAGAACCGTG CCTTCACATT TCAAGGTACG GAACTGGTTG GAAAACGAAA CAGACGCAAG -140
+CTCT
+A
+AACCGTGCCTTCACATTTCAAG
+TCTCGAGGTCGGTTTTCCT
+AAGAACCGTGCCTTCACATTTCAAGGTACGGAACTGGTTGGAAAACGAAACAGACGCAAGAAGCAGAACGAGACTG
ΔGA
ΔG-AGAC
+T3x
ΔGCTGCA-GATGTTTTCC-G
+ATGTTTTCCGTATCTTACGCAACTACAG1x
141- AAGCAGAACG AGACTGCTTT TCAAGCTGCA GATGTTTTCC GTATCTTACG CAACTACAGT GGCAGACAAC -210
+AGCTGCA
+AGAG
+G
ΔAT
ΔAA
ΔG-A
+TG
211- CTCACCTATC ACAAAGAGAG AACAACGCTG TCGGAAAGCTT ACTCACCATT ATTTCTGCC GCAAATCAAA -280
ΔTG
ΔT-CTGGAAGTAG-GGATAACTGTG-AATG2x
281- GAATTACCGA GGATCACTGT CTGGAAGTAG GGATAACTGTG AATGGCCATC AGGCGGATCA GGAGCAACA -350
G. mph1 + A3A (n=26)
1- ATGGGAGGAC CTAGGAGAGA AACAAACAGA GACGAAACAA CAAACAGGAT GGGAGGTCTC GAGGTCGGTT -70
+TT2x
71- TTCCTCTCTC AAGAACCGTG CCTTCACATT TCAAGGTACG GAACTGGTTG GAAAACGAAA CAGACGCAAG -140
GTTTT+TG
TTTTT+TG2x
+TG20x
141- AAGCAGAACG AGACTGCTTT TCAAGCTGCA GATGTTTTCC GTATCTTACG CAACTACAGT GGCAGACAAC -210
211- CTCACCTATC ACAAAGAGAG AACAACGCTG TCGGAAAGCTT ACTCACCATT ATTTCTGCC GCAAATCAAA -280
ΔGA>+TTC
281- GAATTACCGA GGATCACTGT CTGGAAGTAG GGATAACTGTG AATGGCCATC AGGCGGATCA GGAGCAACA -350

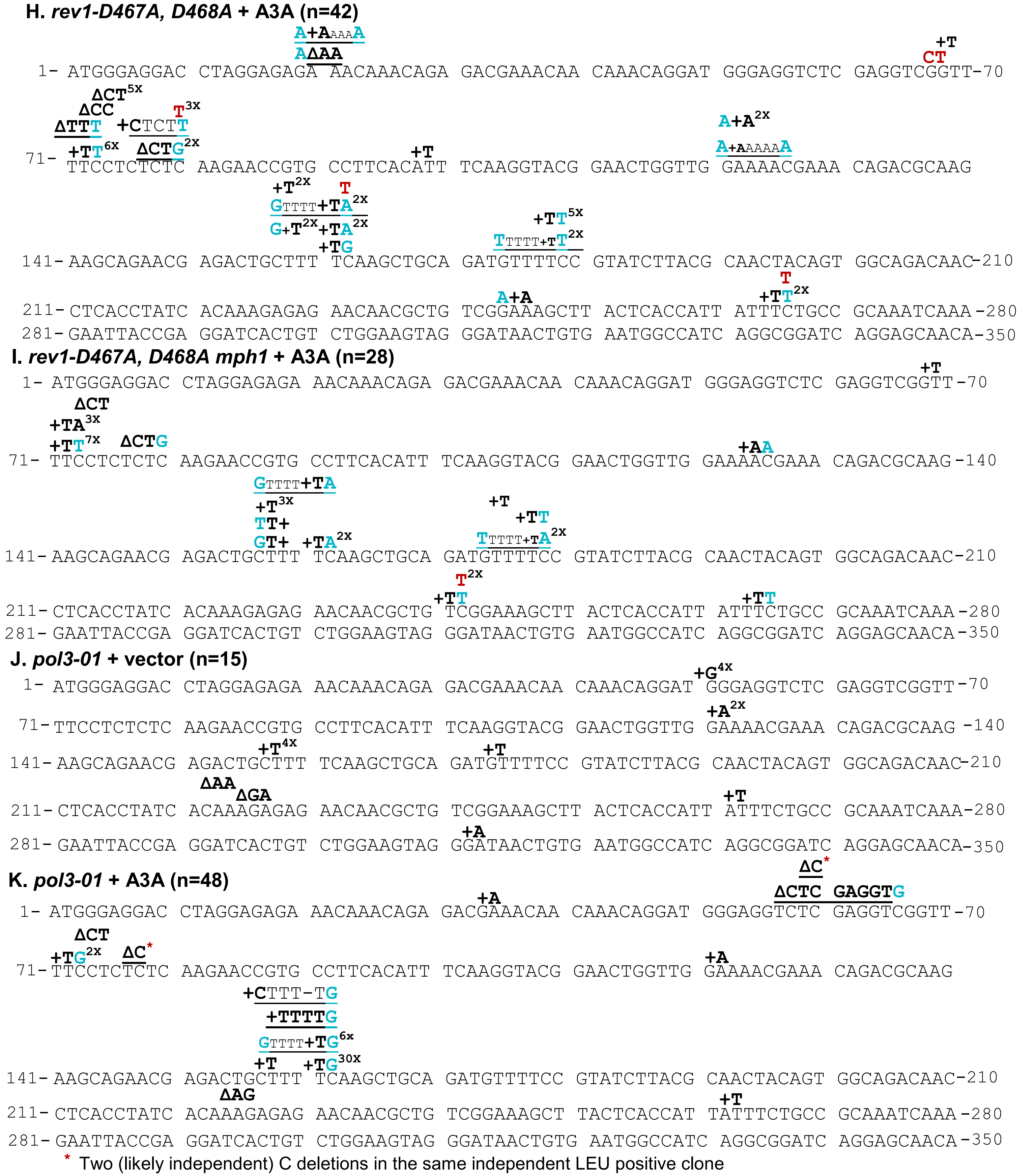

Supplementary Figure 3: The distribution of mutations mapped to the (3n+1) reversion reporter sequence. Mutations are in bold text. Delta (**Δ**) and plus (**+**) symbols indicated bases deleted and inserted bases, respectively. Base pair substitutions are in aqua blue for simple and complex events. Base pair substitutions that are likely passenger mutations (distant from the indel events in the same sequenced sample) are in dark red font. Arrows indicate positions of large insertions. Unmutated bases within complex mutations are normal (not bold) font. (n) denotes the number of sequenced events. Complex events are depicted as joined text string above the reference sequence. Underlining was used for complex events with multiple mutations to clearly denote single mutation events. Superscripts denote multiples of the same mutation.

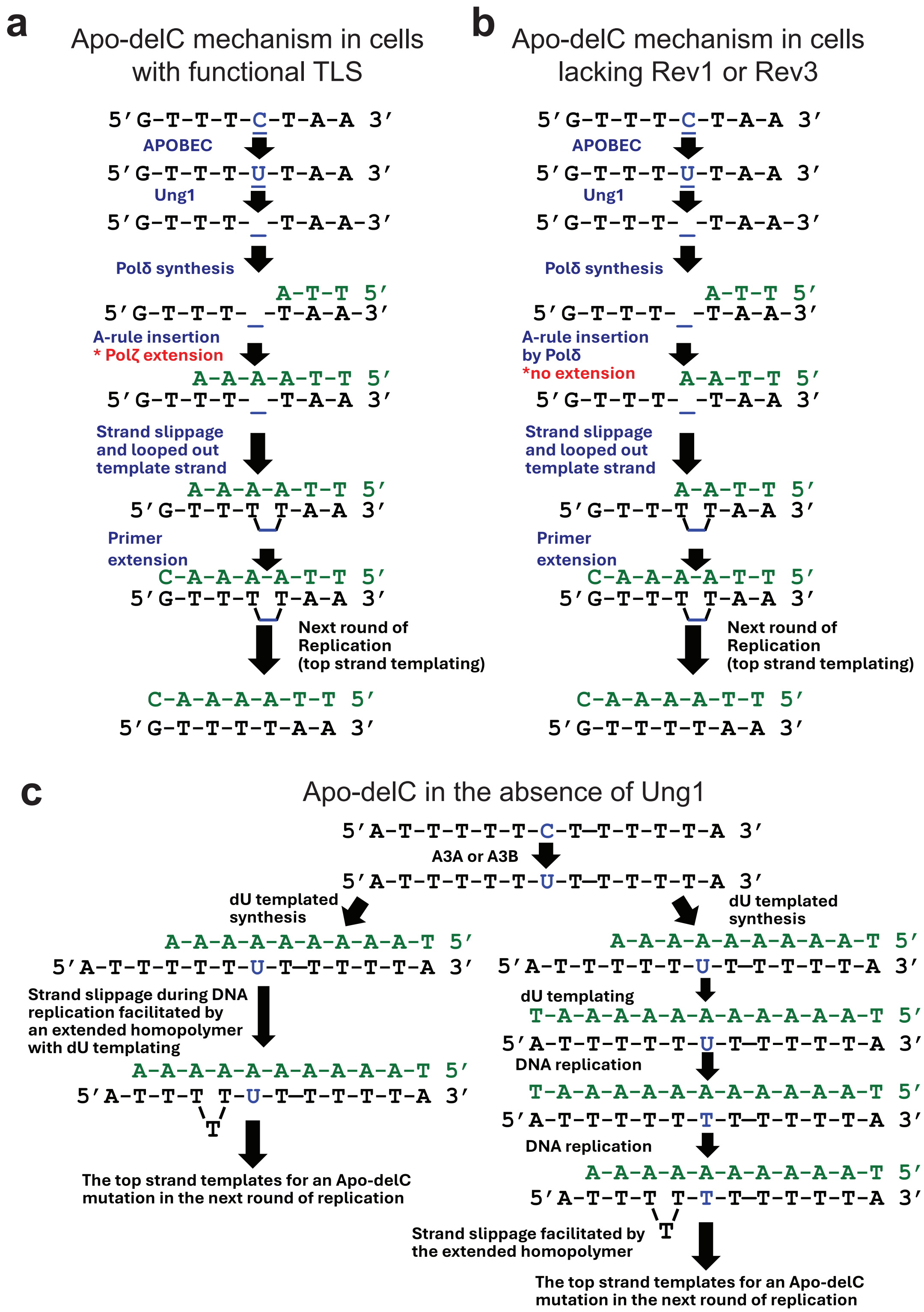

Supplementary Figure 4: Proposed mechanisms for Apo-delC mutations. Black and green fonts represent the lagging strand template and newly synthesized DNA during DNA replication, respectively. The base corresponding to the cytidine deaminated by A3BA/A3B is in blue. **(a)** Apo-delC mutations in wild-type yeast expressing A3A: A-rule insertion, Polζ extension, template slippage, and Polζ extension. **(b)** Apo-delC mutations in Rev1 or Rev3 deficient yeast expressing A3A: A-rule insertion, template slippage, and extension (likely by Polδ). **(c)** Apo-delC mutations observed in yeast WGS in strains lacking Ung1, which must occur independently of abasic site bypass: slippage events, which increase exponentially in frequency as homopolymer lengths increase, are increased due to homopolymer perfection caused by dU templated synthesis.

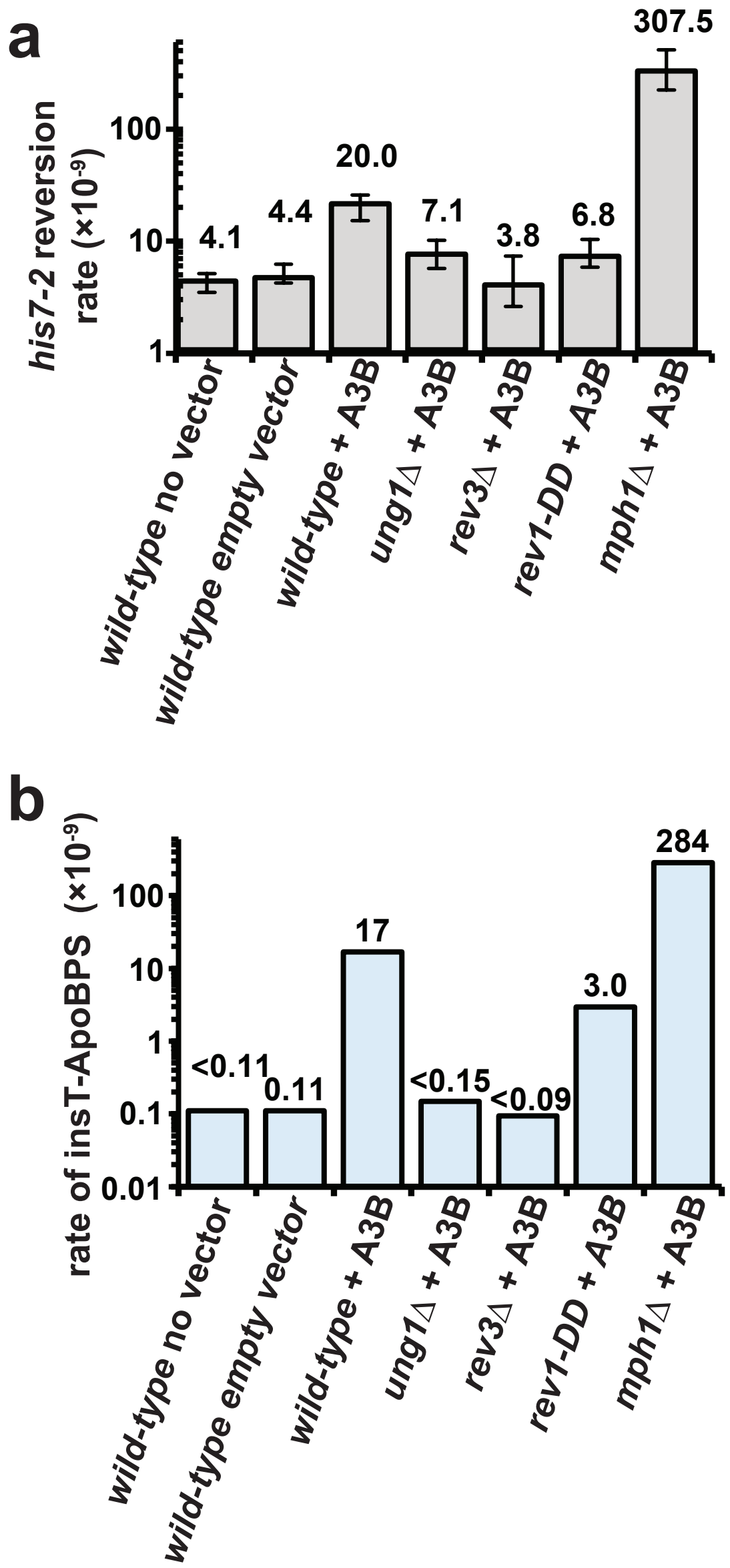

Supplementary Figure 5: Measurement of insT-ApoBPS mutations at *his7-2*. (a) Observed *his7-2* reversion rates, which includes all mutation types. Error bars represent 95% confidence intervals and data labels display the numeric value of the mutation rate ($\times 10^{-8}$). **(b)** Rates for insT-ApoBPS mutations, which were calculated by multiplying the reversion rate by the fraction of the mutation events that were insT-ApoBPS events. Less than values (<) indicated no insT-ApoBPS events were observed; these values were calculated using a frequency of one event.

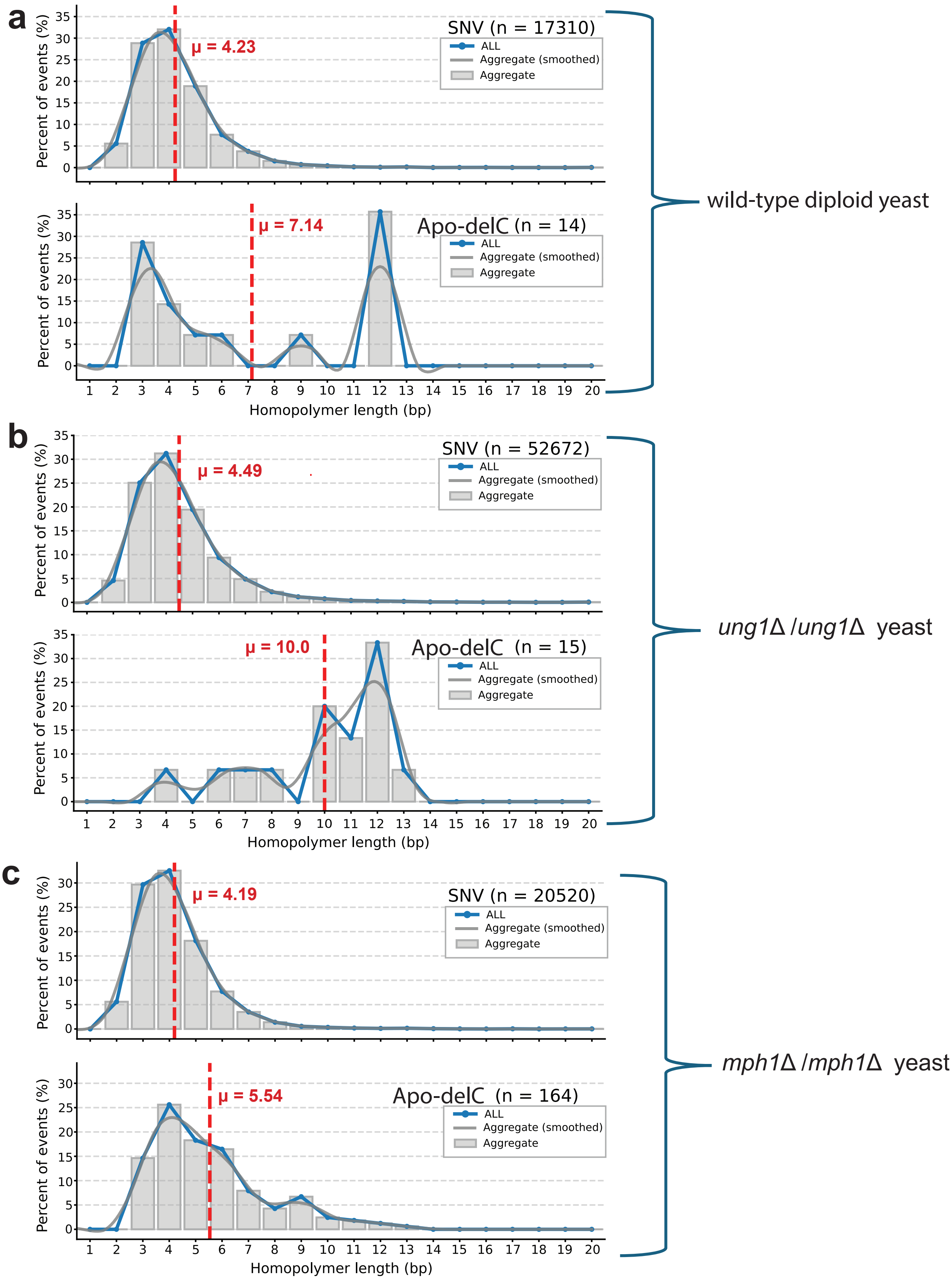

Supplementary Figure 6: Perfected Homopolymer length of Apo-delC mutations. The top graph in each panel shows the homopolymer length that would be generated C>T mutations at all C>T and C>G mutations in TC motifs identified in yeast WGS. The bottom graph shows the T homopolymer lengths that would be generated by deamination of dC to dU or C>T mutations at the genomic coordinates of Apo-delC mutations. **(a)** Data from wild-type diploid yeast. **(b)** Data from *ung1Δ/ung1Δ* diploid yeast. **(c)** Data from *mph1Δ/mph1Δ* diploid yeast.

connected T homopolymer

```
                          +T   T
tcttcattagagaccggttgatattggttttCaatgctgaaaaaggggaactgcacag
```

disconnected D (D= A, C, or G nucleotides) homopolymer

```
                          +A   G
ataaataataacaacgaatataaatacaaatCgaaagaattcagattgaattcaatat
```

disconnected T homopolymer

```
                        +T     T
atgtccattagctgtgtaactggaattttatCaggggtggcattgatttttttgttaa
```

Supplementary Figure 7: Examples of connected vs. disconnected ins-ApoBPS mutations from yeast WGS. Mutations are shown in bold font above the reference sequence. The BPS in each ins-ApoBPS is in blue font and the insertion is denoted by a plus symbol adjacent to the nucleotide inserted, in black font above the homopolymer where the insertion occurred.

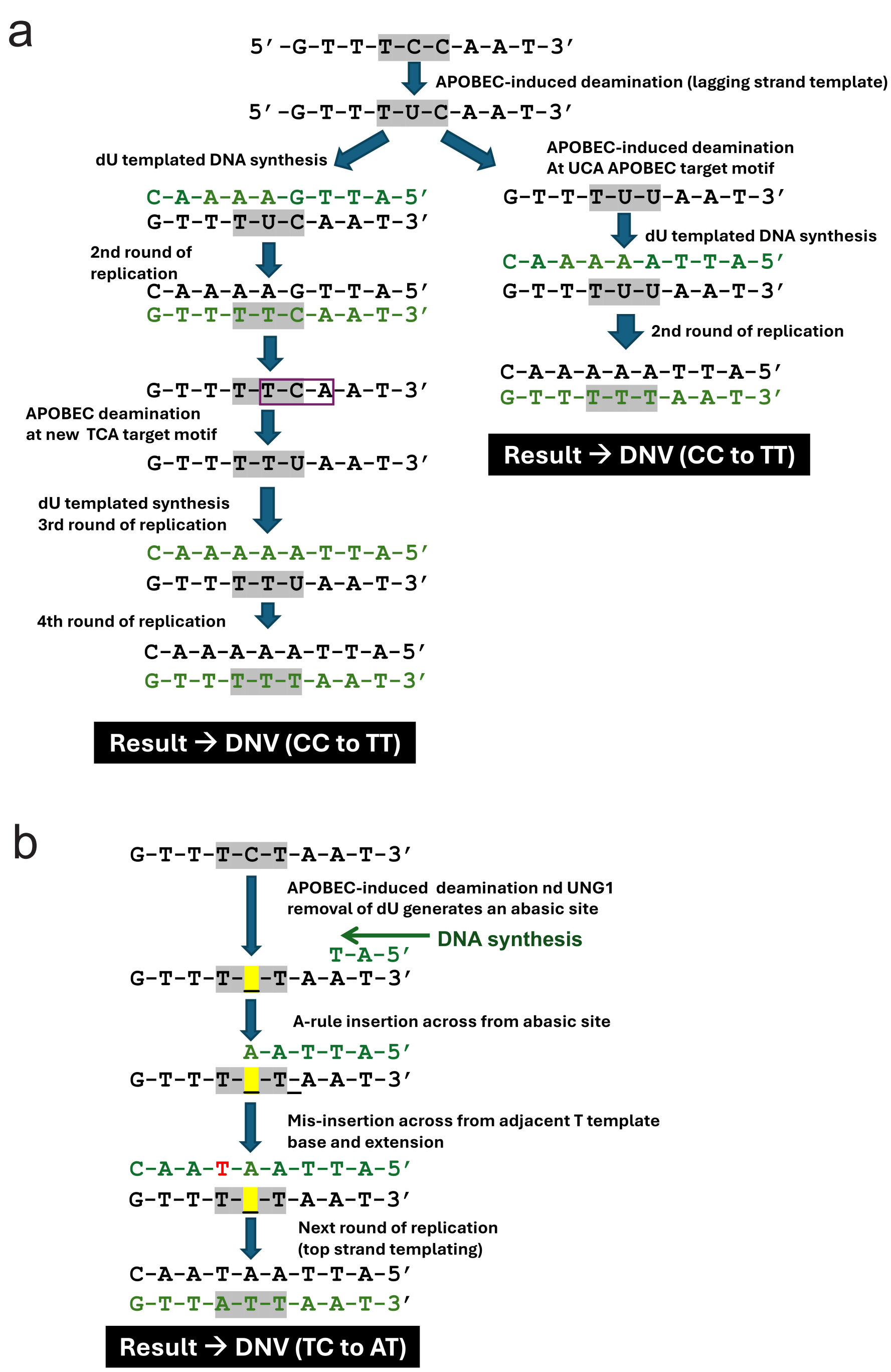

Supplementary Figure 8: Proposed mechanisms for APOBEC-induced DNVs. The DNA sequence in black font represents the lagging strand template in each panel. Newly synthesized DNA is in green font. The location of the original APOBEC target motif is highlighted grey throughout. Mismatched base pairs are in red font and abasic sites are highlighted yellow. **(a)** CC to TT DNVs are increased in cells lacking Ung1/UNG2 and likely occur due to dU templating. Deamination of the 1st C in TCC motifs creates a new target motif TUC or TTC following subsequent rounds of DNA replication. **(b)** TC to AT/CT/GT DNVs are reduced in cells lacking Ung1/UNG2 and increased in strains lacking Mph1, thus they likely occur during TLS bypass of abasic sites.

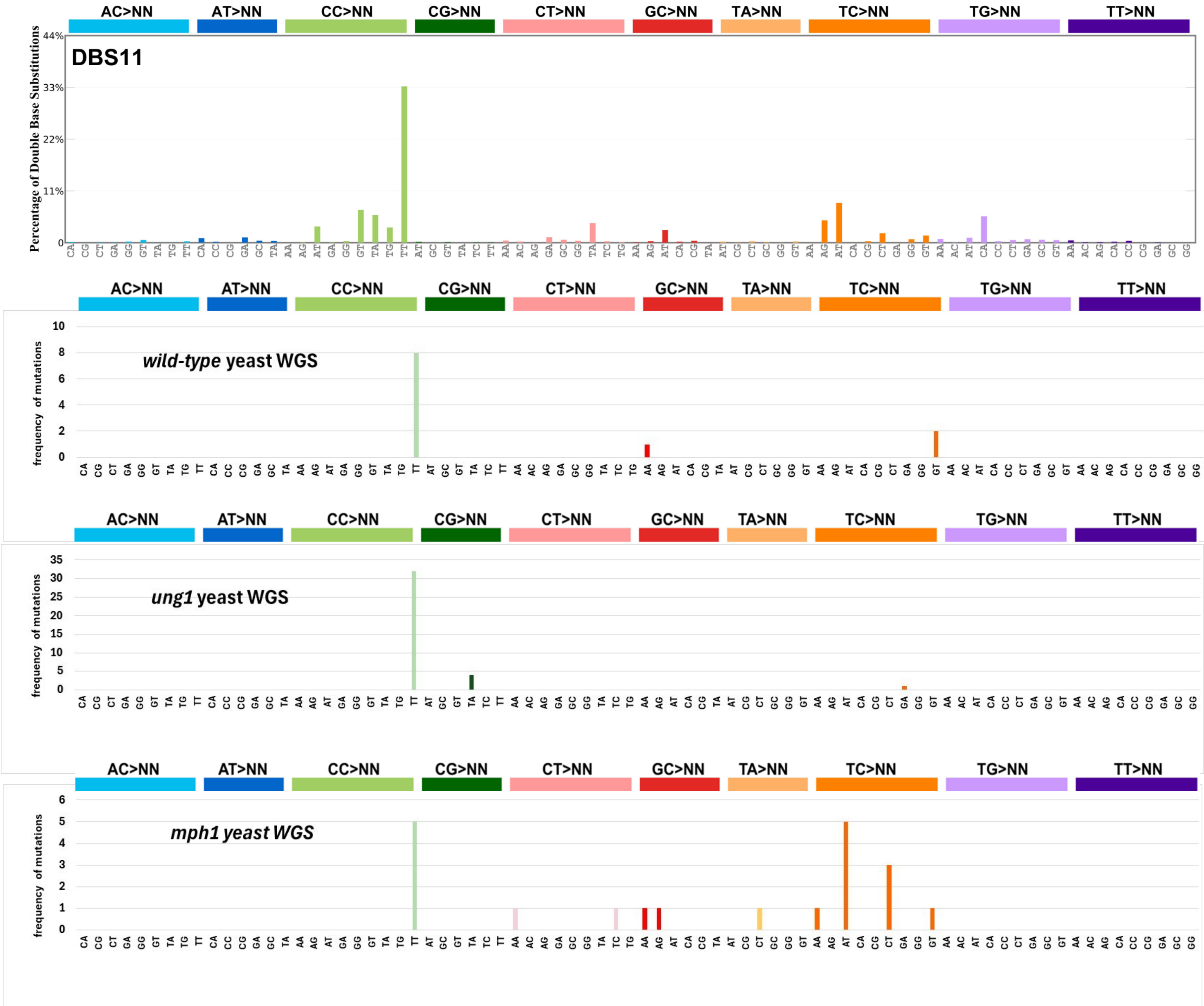

Supplementary Figure 9: APOBEC-induced DNV / DBS signatures in yeast. Top panel is DBS11 from https://cancer.sanger.ac.uk/signatures/dbs/dbs11/. The bottom three panels are the frequency of each DBS mutation type observed in yeast WGS. CC to TT and TC to AT/CT/GT components of DBS11 are increased in Ung1 and Mph1 deficient yeast, respectively.

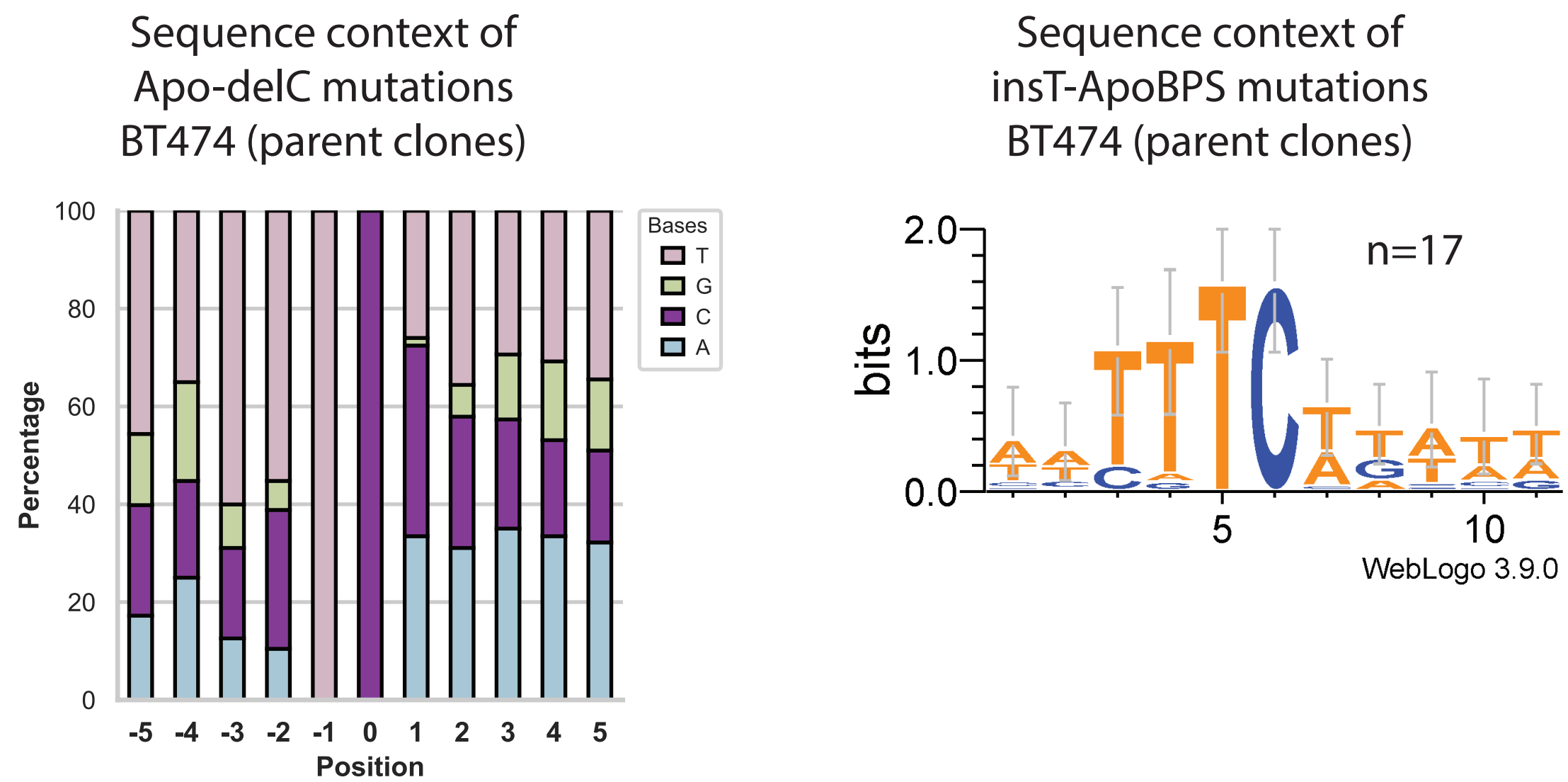

Supplementary Figure 10: Sequence context of Apo-delC and insT-ApoBPS in human cell lines. (a) Sequence context of Apo-delC mutations in BT474 clones. **(b)** Sequence context of insT-ApoBPS generated using Weblogo(2). Sequence contexts are centered on the APOBEC3 target C.

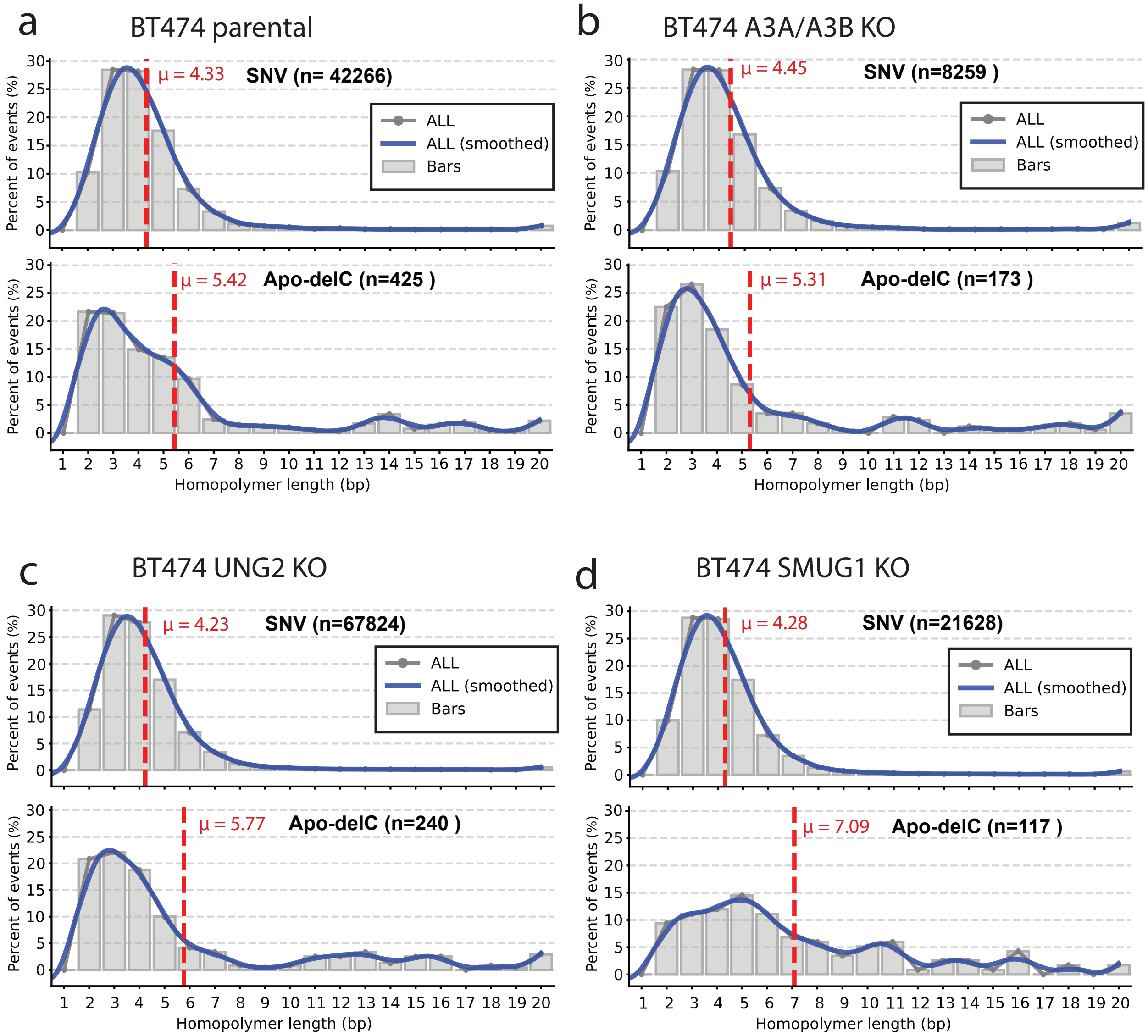

Supplementary Figure 11: Perfected Homopolymer length of Apo-delC mutations in BT474 derived cell lines. The top graph in each panel shows the homopolymer length that would be generated by C>T mutations at all C>T and C>G mutations in TC motifs identified by WGS. The bottom graph shows the T homopolymer lengths that would be generated by deamination of dC to dU or C>T mutations at the genomic coordinates of Apo-delC mutations. **(a)** BT474 parental cells. **(b)** BT474 A3A/A3B KO. **(c)** BT474 UNG2 KO. **(d)** SMUG1 KO.

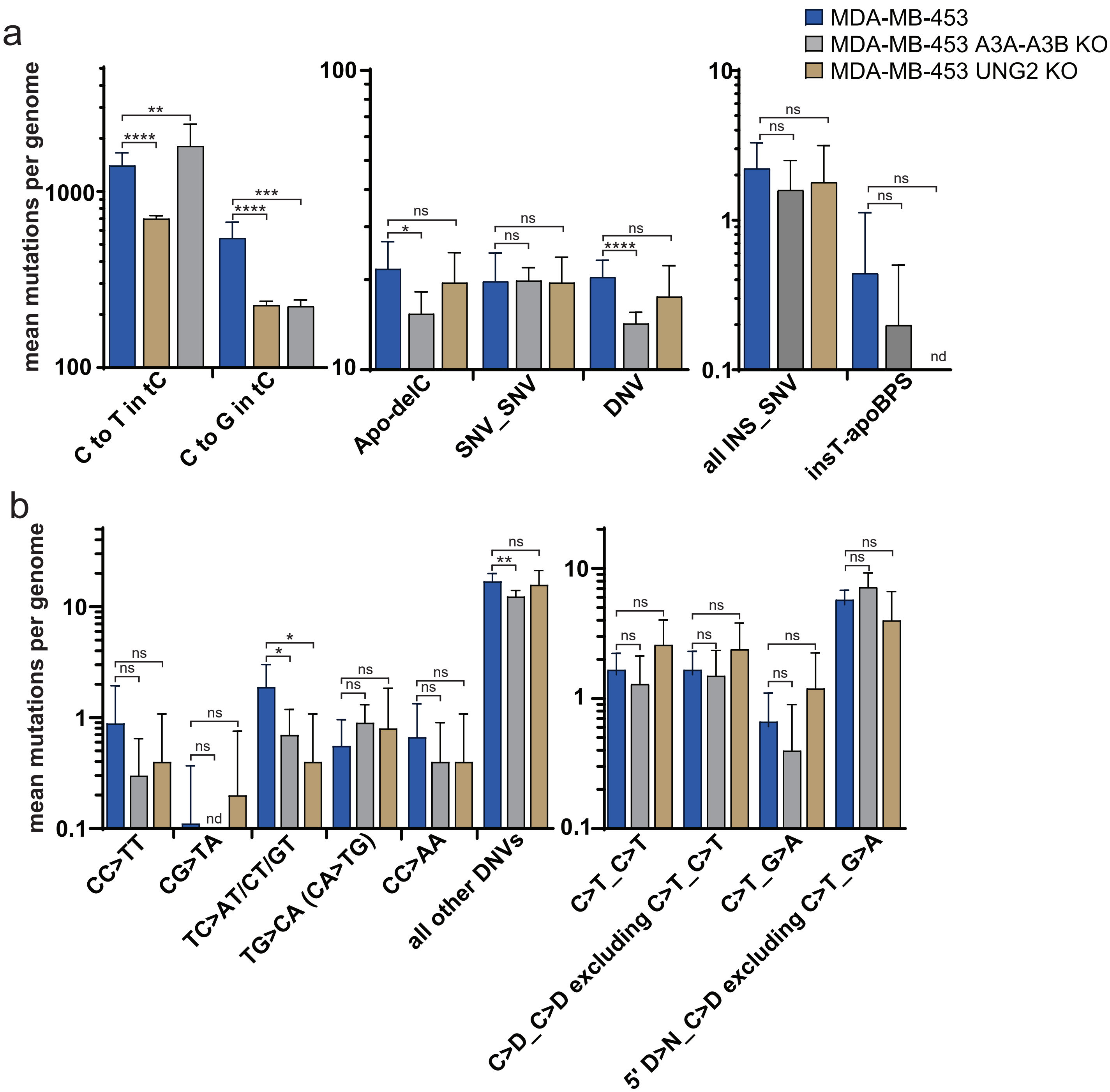

Supplementary Figure 12: Mean mutations per genome in MDA-MB-453 cells and MDA-MB-453 derived knockout cell lines. Analysis of mutations accumulated in clonal outgrowth clones from(3). Not detected (ND). Statistical evaluation of comparisons by Pairwise Mann-Whitney U tests (**** p=0.0001, *** p=0.001, ** p=0.01, * p=0.05, ns = non-significant). **(A)** Major classes of mutations. **(B)** Subclasses of DNVs and complex SNV-SNV events.

Dataset S1 (separate Excel file). List of all yeast mutations from mutation reporters and summary of mutation spectra.

Dataset S2 (separate Excel file). All yeast mutation rates observed with mutation reporters.

Dataset S3 (separate Excel file). List of all mutations from yeast whole genome sequencing.

Dataset S4 (separate Excel file). Mutation frequencies from yeast WGS by genotype and sample.

Dataset S5 (separate Excel file). Analysis of SNV-SNV events: intergenic vs. transcribed strand vs. non-transcribed strand.

Dataset S6 (separate Excel file). List of all complex mutations from whole genome sequencing human cell lines.

Dataset S7 (separate Excel file). List of all non-complex mutations from BT474 cells.

Dataset S8 (separate Excel file). List of all non-complex mutations from BT474 KO cell lines.

Dataset S9 (separate Excel file). List of all non-complex mutations from MDA-MB-453 and its derived KO cell lines.

Dataset S10 (separate Excel file). Mutation frequencies from human cell line WGS for complex events and DNVs with phasing and additional data.

Dataset S11 (separate Excel file). Mutation frequencies from human cell line WGS for all mutation types.

Dataset S12 (separate Excel file). Mutation frequencies from breast cancer tumors.

Dataset S13 (separate Excel file). List of oligos and plasmids.

Dataset S14 (separate Excel file). List of yeast strains used in this study.

SI References

1. H. T. Tran, J. D. Keen, M. Kricker, M. A. Resnick, D. A. Gordenin, Hypermutability of homonucleotide runs in mismatch repair and DNA polymerase proofreading yeast mutants. *Mol Cell Biol* **17**, 2859-2865 (1997).
2. G. E. Crooks, G. Hon, J. M. Chandonia, S. E. Brenner, WebLogo: a sequence logo generator. *Genome Res* **14**, 1188-1190 (2004).
3. M. Petljak *et al.*, Mechanisms of APOBEC3 mutagenesis in human cancer cells. *Nature* **607**, 799-807 (2022).
